# Supplementary figures and images for: Exploring main soil drivers of vegetation succession in abandoned croplands of Minqin Oasis, China
Source: PeerJ. 2024 Jul 5;12:e17627. doi: 10.7717/peerj.17627 (PMC11229685; doi:10.7717/peerj.17627)

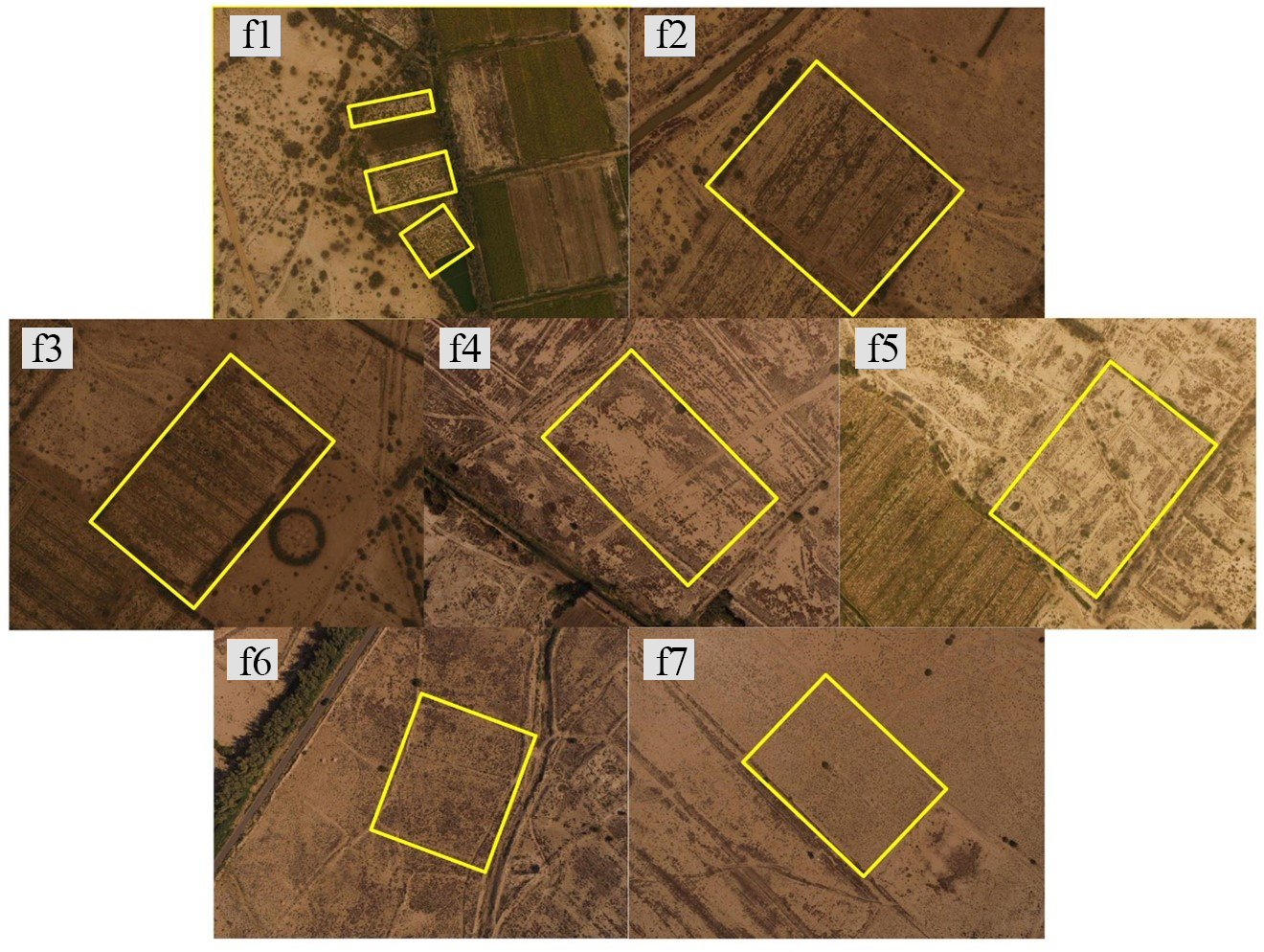

Supplement: Supplemental Information 1 — Seven abandoned croplands are labelled f1 to f7 in the Minqin Oasis. Years since abandonment appear in parentheses following labels, f1 (1a), f2 (6a), f3 (7a), f4 (8a), f5 (9a), f6 (20a) and, f7 (29a). [file peerj-12-17627-s001.png]
