# Supplementary material for: Exploring main soil drivers of vegetation succession in abandoned croplands of Minqin Oasis, China
Source: PeerJ. 2024 Jul 5;12:e17627. doi: 10.7717/peerj.17627 (PMC11229685; doi:10.7717/peerj.17627)

**File specification**: There are four files in the folder.

1. “1-1read me-metadata.docx” is an instruction file.

2. “1-2species-Twinspan.xls” is a data file. The data format is required for the software of Pcord 5.0. On the top left corner, it is the number of plots and species. The capital letter “Q” doesn’t have any specific meaning, but the number of “Q” is equal to the number of species. The main data is based on a 33 column × 21 row (33 species × 21 plots) matrix data. The species names are as follows in the table.

|  | species name |  | species name |
| --- | --- | --- | --- |
| sp1 | *Halogeton glomeratus* | sp17 | *Peganum harmala* |
| sp2 | *Kochia scoparia* | sp18 | *Glycyrrhiza uralensis* |
| sp3 | *Convolvulus arvensis* | sp19 | *Achnatherum splendens* |
| sp4 | *Atriplex centralasiatica* | sp20 | *Suaeda prostrata* |
| sp5 | *Chloris virgata* | sp21 | *Setaria viridis* |
| sp6 | *Suaeda glauca* | sp22 | *Cardaria chalepensis* |
| sp7 | *Chenopodium album* | sp23 | *Nitraria tangutorum* |
| sp8 | *Phragmites australis* | sp24 | *Tamarix hispida* |
| sp9 | *Mulgedium tataricum* | sp25 | *Cirsium setosum* |
| sp10 | *Cynanchum sibiricum* | sp26 | *Leymus secalinus* |
| sp11 | *Peganum nigellastrum* | sp27 | *Nitraria sibirica* |
| sp12 | *Lycium chinense* | sp28 | *Kalidium foliatum* |
| sp13 | *Euphorbia humifusa* | sp29 | *Limonium aureum* |
| sp14 | *Echinochloa crusgalli* | sp30 | *Bassia dasyphylla* |
| sp15 | *Tamarix chinensis* | sp31 | *Acroptilon repens* |
| sp16 | *Lycium ruthenicum* | sp32 | *Cynanchum chinense* |
|  |  | sp33 | *Reaumuria songarica* |

3. “1-3TWINSPAN-result.txt” is a result file.

4. “1-4TWINSPAN-workflow.doc” is a workflow file, and it shows us how to perform the TWINSPAN analysis in the software of Pcord 5.0.


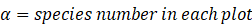

Supplement: Supplemental Information 2 — The metadata, raw data, analysis workflow, and result of: 1TWINSPAN-workflow, 2CCA-workflow, 3GAM-species response curves-workflow, and 4K-W test of plant diversity-workflow. [file peerj-12-17627-s002.zip › workflow/1TWINSPAN-workflow/1-1read me-metadata.docx]
