# Supplementary material for: Exploring main soil drivers of vegetation succession in abandoned croplands of Minqin Oasis, China
Source: PeerJ. 2024 Jul 5;12:e17627. doi: 10.7717/peerj.17627 (PMC11229685; doi:10.7717/peerj.17627)

**File specification**: There are seven files in the folder.

1. “2-1read me-metadata.docx” is an instruction file.

2. “2-2species data.xlsx” is a data file. The data format is required for the software of Canoco 5.0. The main data is based on a 33 column × 21 row (33 species × 21 plots) matrix data. The species names are as follows in the table.

|  | species name |  | species name |
| --- | --- | --- | --- |
| sp1 | *Halogeton glomeratus* | sp17 | *Peganum harmala* |
| sp2 | *Kochia scoparia* | sp18 | *Glycyrrhiza uralensis* |
| sp3 | *Convolvulus arvensis* | sp19 | *Achnatherum splendens* |
| sp4 | *Atriplex centralasiatica* | sp20 | *Suaeda prostrata* |
| sp5 | *Chloris virgata* | sp21 | *Setaria viridis* |
| sp6 | *Suaeda glauca* | sp22 | *Cardaria chalepensis* |
| sp7 | *Chenopodium album* | sp23 | *Nitraria tangutorum* |
| sp8 | *Phragmites australis* | sp24 | *Tamarix hispida* |
| sp9 | *Mulgedium tataricum* | sp25 | *Cirsium setosum* |
| sp10 | *Cynanchum sibiricum* | sp26 | *Leymus secalinus* |
| sp11 | *Peganum nigellastrum* | sp27 | *Nitraria sibirica* |
| sp12 | *Lycium chinense* | sp28 | *Kalidium foliatum* |
| sp13 | *Euphorbia humifusa* | sp29 | *Limonium aureum* |
| sp14 | *Echinochloa crusgalli* | sp30 | *Bassia dasyphylla* |
| sp15 | *Tamarix chinensis* | sp31 | *Acroptilon repens* |
| sp16 | *Lycium ruthenicum* | sp32 | *Cynanchum chinense* |
|  |  | sp33 | *Reaumuria songarica* |

3. “2-3soil data.xlsx” is also a data file. The main data is based on a 9 column × 21 row (9 soil factors × 21 plots) matrix data. The units of soil factors are as follows in the table.

| abbreviation | name | units |
| --- | --- | --- |
| SOM | soil organic matter | % |
| AP | available phosphorus | mg/100g soil |
| pH | pH value | - |
| EC | electrical conductivity | μm/cm |
| TN | total nitrogen | % |
| SSW | saturated soil water content | % |
| SFC | soil field capacity | % |
| TS | total salinity | % |
| Year | years of land abandonment | a |

4. “2-4CCA-workflow.docx” is a workflow file, and it shows us how to perform the CCA analysis in the software of Canoco 5.0.

5. “2-5Simple and Conditional term effects workflow.docx” is a workflow file, and it shows us how to get the parameters of simple and conditional term effects in the software of Canoco 5.0.

6. “2-6CCA-result.docx” is a result file of CCA analysis.

7. “2-7Simple and Conditional term effects-result.docx” is a result file of Simple and Conditional term effects analysis.


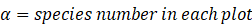

Supplement: Supplemental Information 2 — The metadata, raw data, analysis workflow, and result of: 1TWINSPAN-workflow, 2CCA-workflow, 3GAM-species response curves-workflow, and 4K-W test of plant diversity-workflow. [file peerj-12-17627-s002.zip › workflow/2CCA-workflow/2-1read me-metadata.docx]
