# Supplementary material for: Exploring main soil drivers of vegetation succession in abandoned croplands of Minqin Oasis, China
Source: PeerJ. 2024 Jul 5;12:e17627. doi: 10.7717/peerj.17627 (PMC11229685; doi:10.7717/peerj.17627)

CCA-workflow

1.
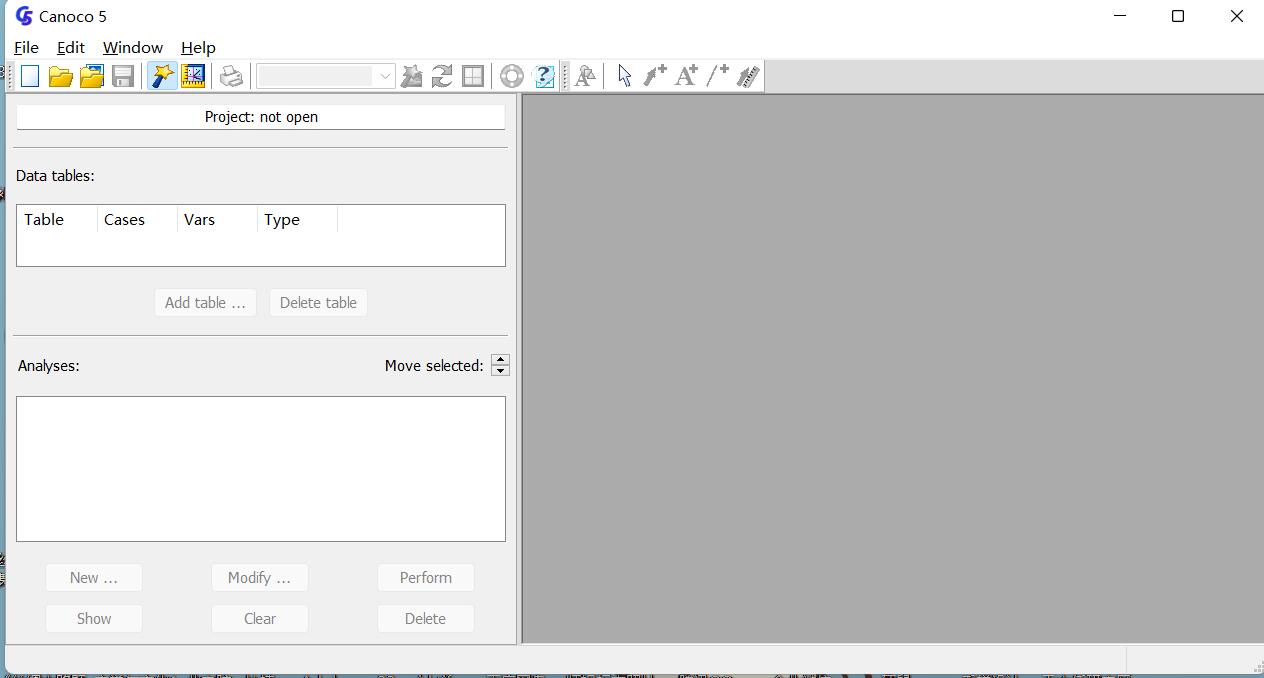
Open the software of Canoco 5.0, and the following window is open.
2. Input raw data
3. Import two matrix data files “species1.xlsx” and “soil.xlsx” into the software of Canoco 5 by selecting Import project | from Excel.


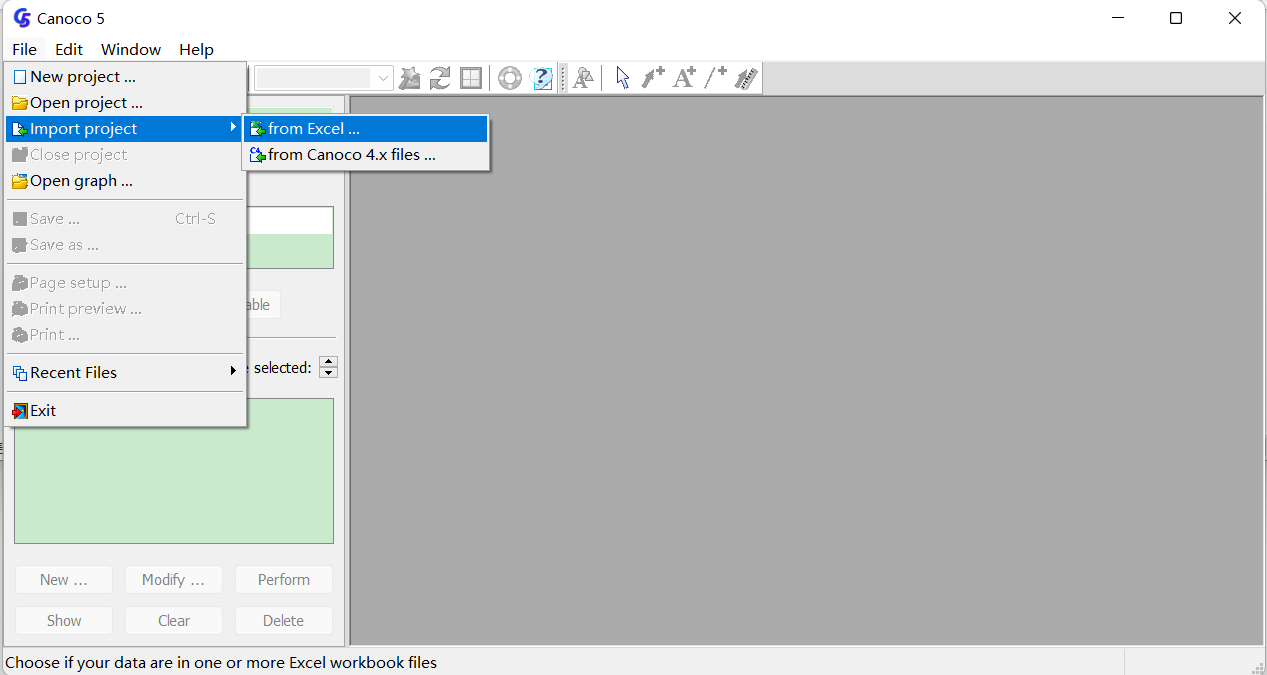


1. At the first page of the import wizard, click the *Add files* button, locate and select the “species1.xlsx” and “soil.xlsx” spreadsheet files, then click the *Next* button.


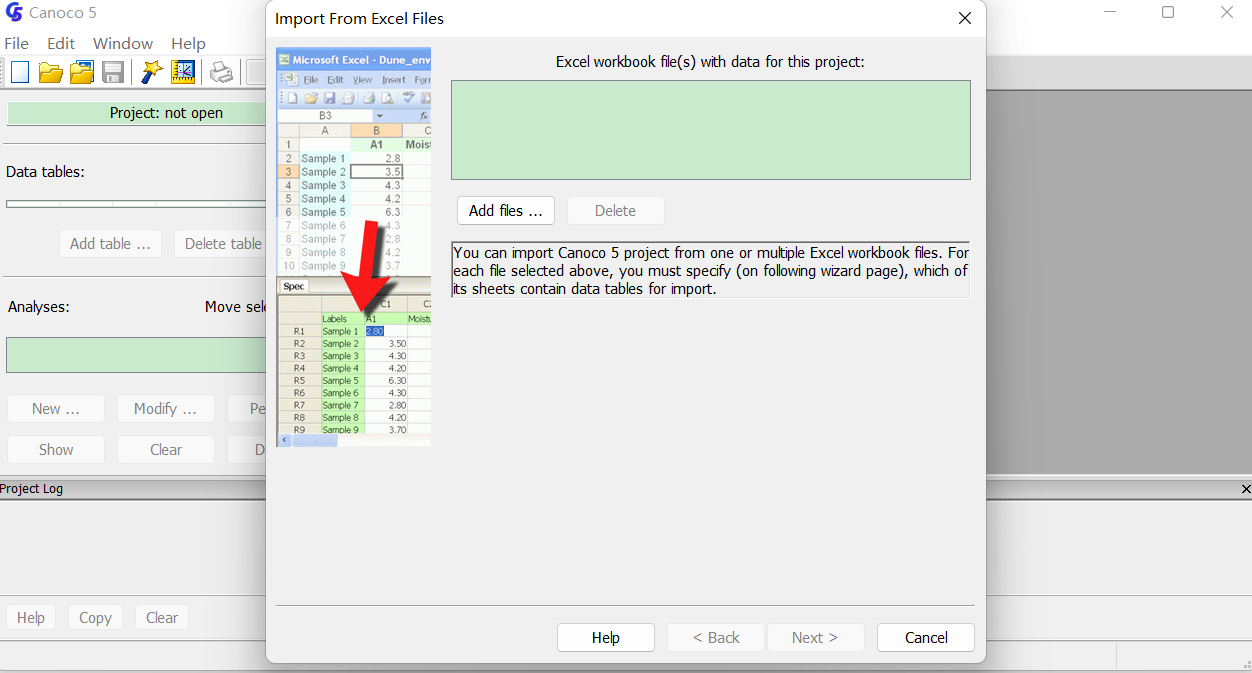


(3) On the following page, the first data-table “species1.xlsx” is marked as compositional data, type “sp” as the *Table name.* The other options can be left at their default values, and press the *Next* button.


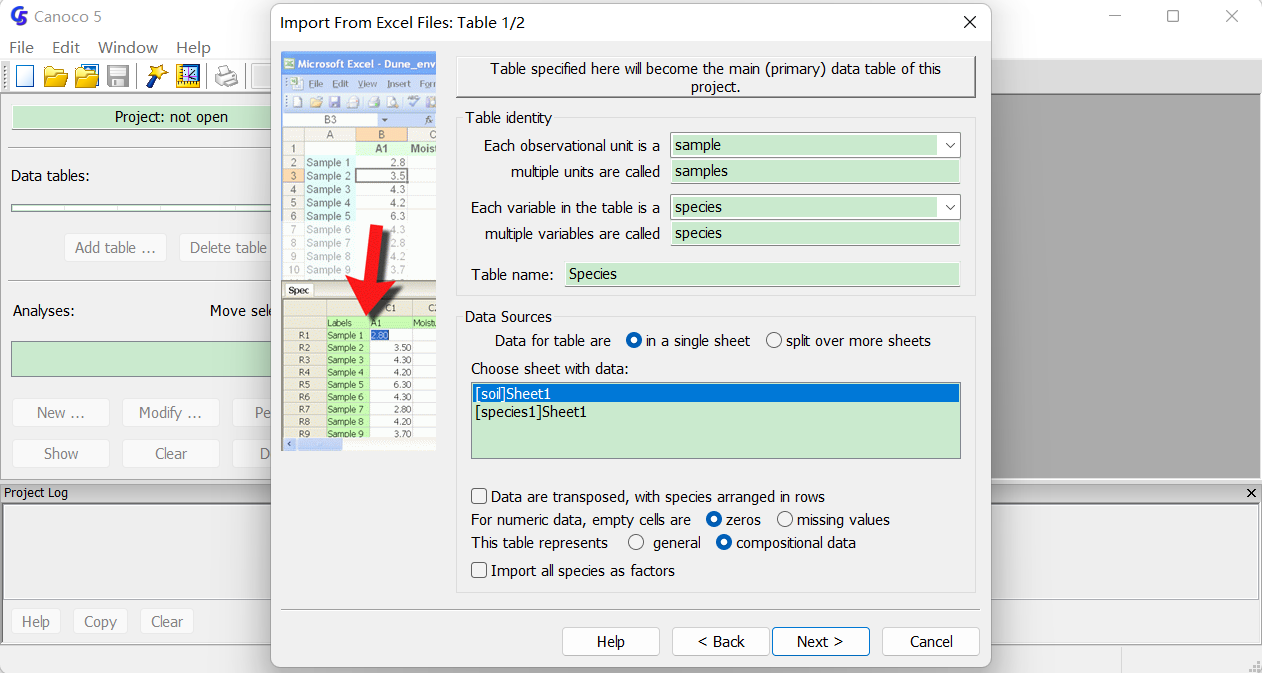


(4) Preview the data range found in the Excel sheet by the import wizard, and click the *Next* button again.


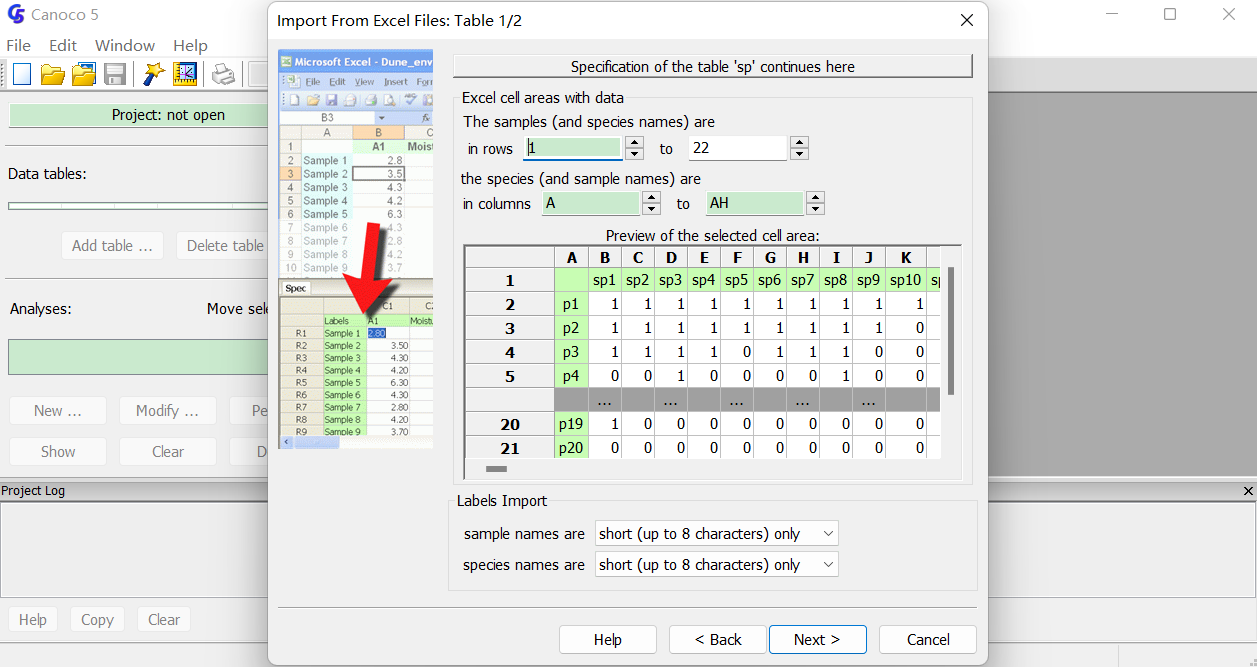


(5) This wizard brings you to specify the second data-table. Select the “soil.xlsx” spread sheet as the “general” data source, type “env” as the *Table name*, then press the *Next* button.


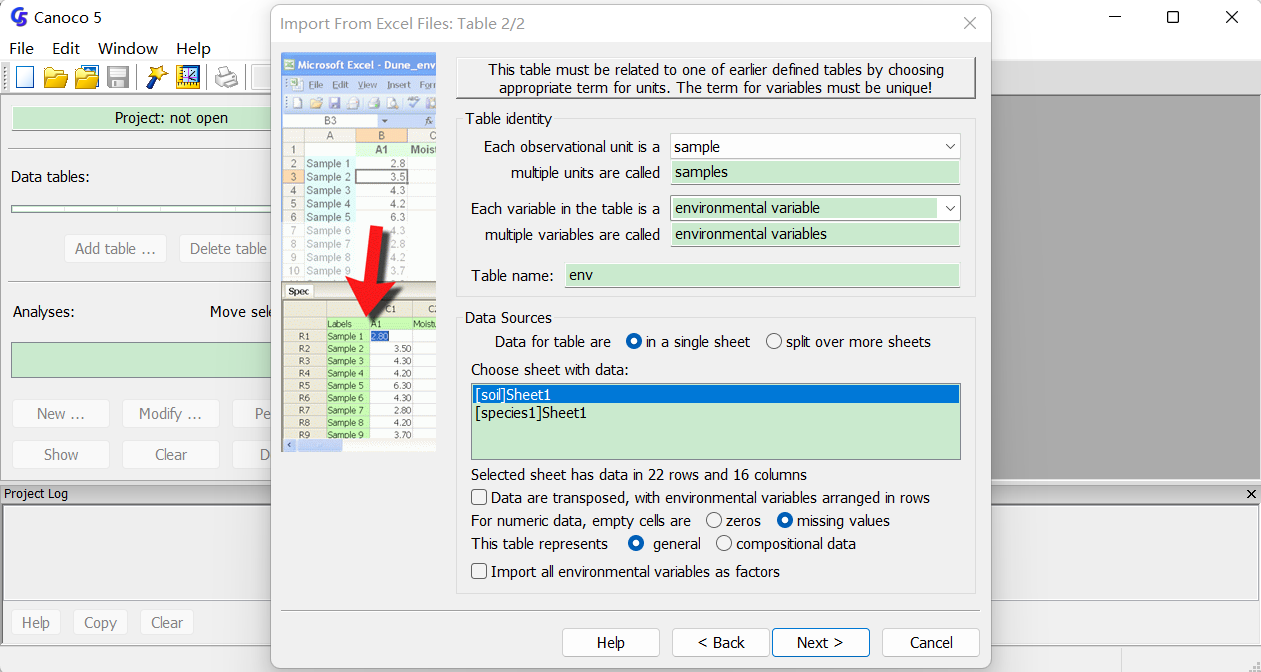


(6) review the selected spreadsheet area with data and close the wizard with the *Finish* button.


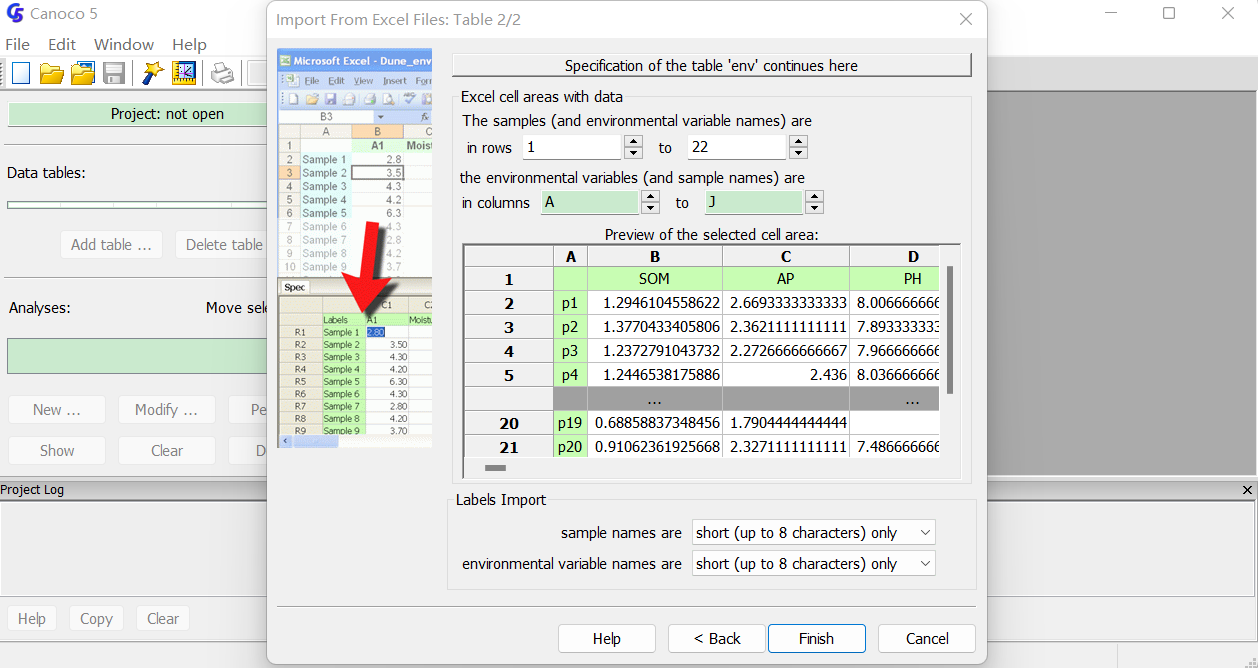


1. perform the process of CCA analysis
2. Select analysis-type (*constrained ordination of species, using all environmental variables*) and click the *Yes* button.


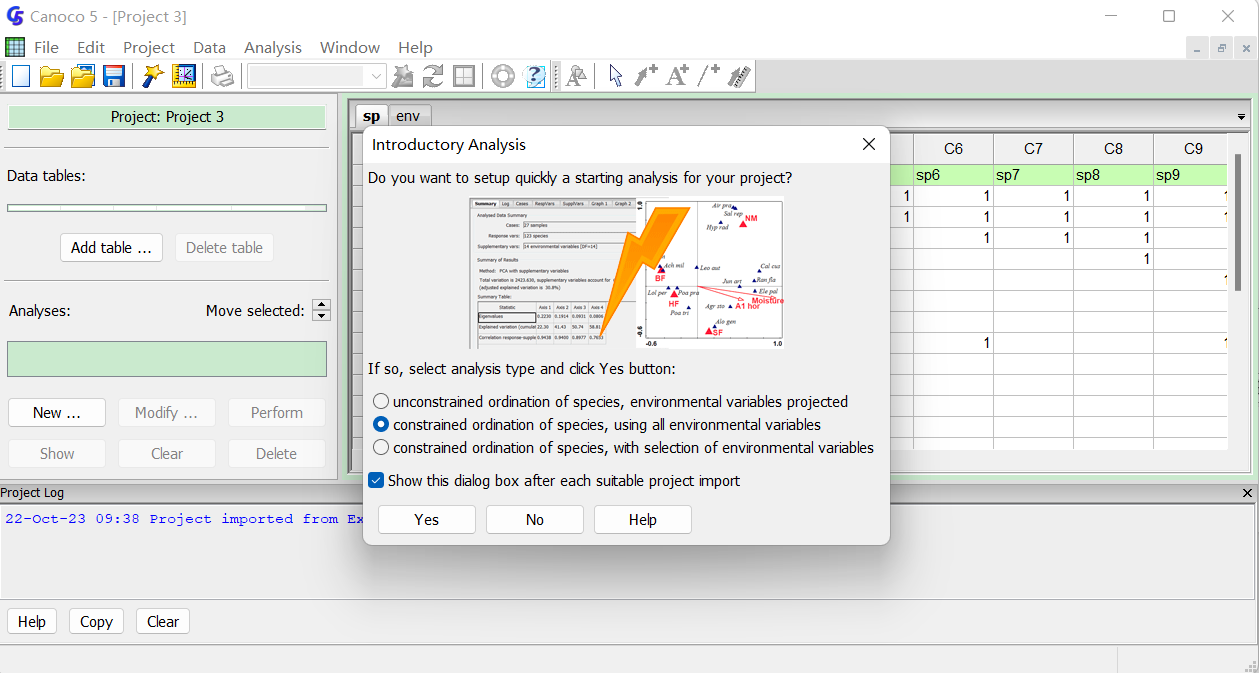


(2) At the dialog box, select *Downweight rare species* and keep the other options unchanged. Click the Next button.


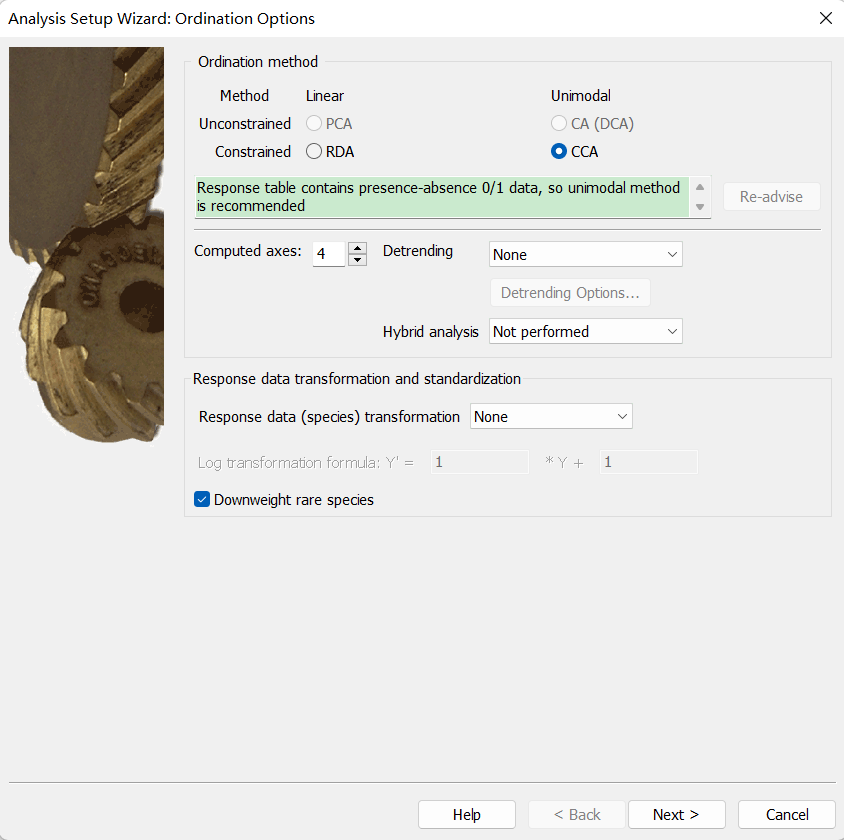


(3) In this dialog box, select *Both above tests performed* and type “9999” as Number of permutations, then press the *Next* button and close the setup wizard on the following page with the *Finish* button.


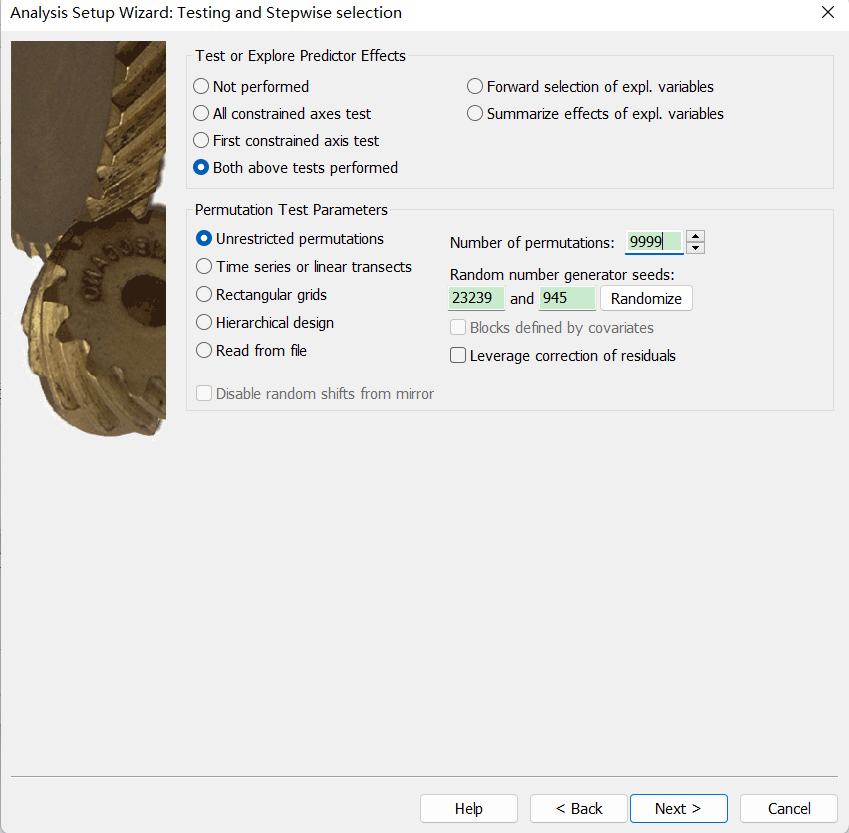


1. Preview the detail results.
2. Select Edit | Settings | Canoco5 options, and a dialog box is shown.


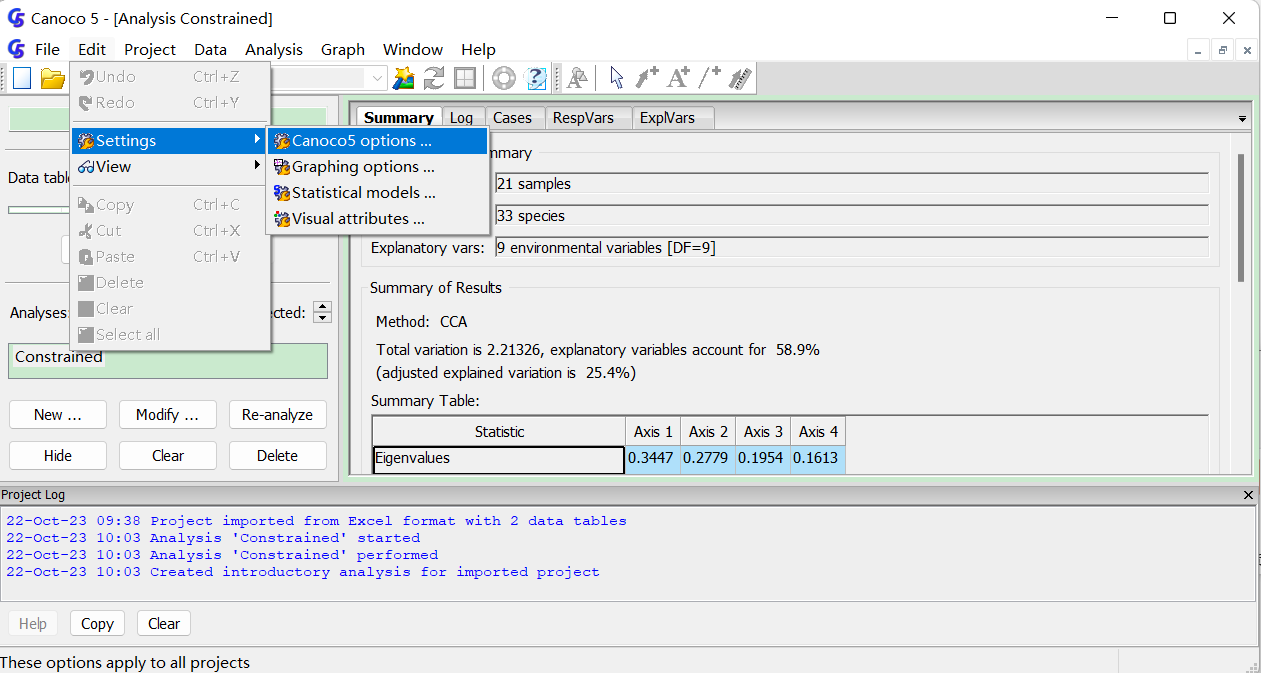


1. In *General* options, uncheck the second option (*Show brief version of notebooks with analysis results*) and click *OK* button.


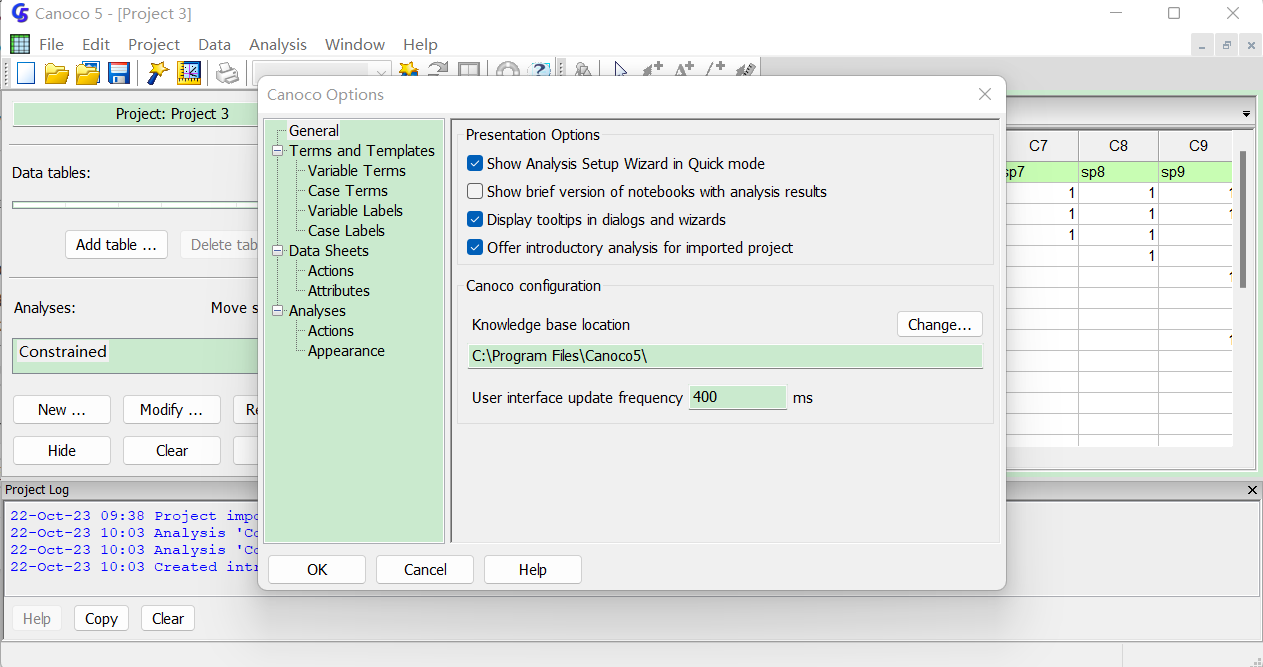


1. The detail results are shown.


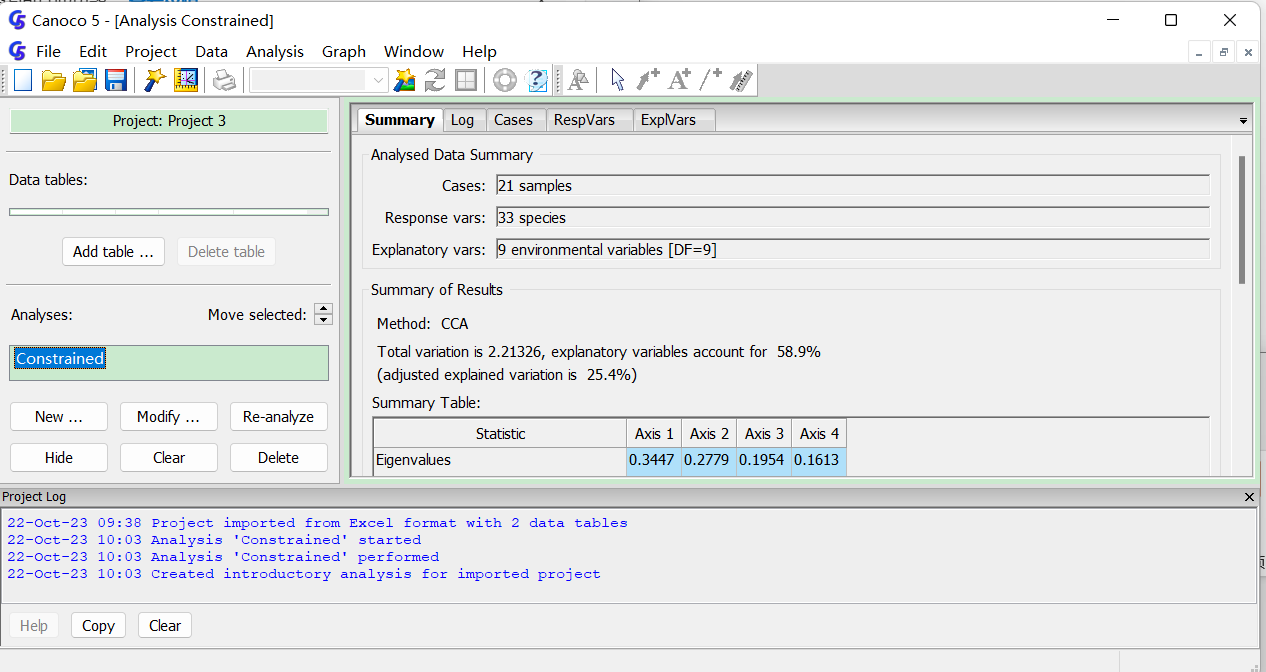


1. Graphs
2. Define classifications of plots from TWINSPAN analysis results with select Project | Classifications | of samples,


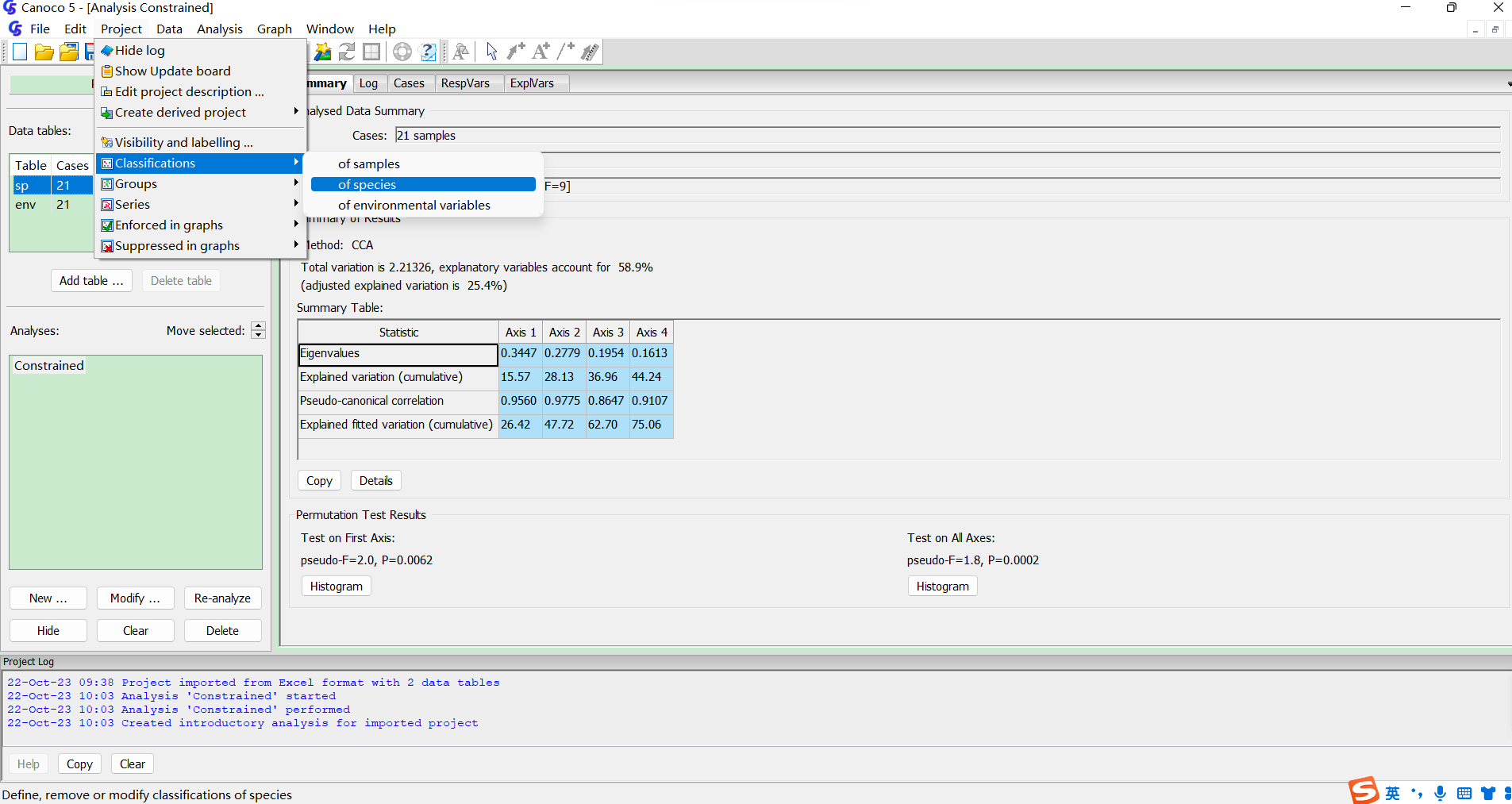


1. At the Classification Manager dialog box, select *By Selection* button to create a new classification.


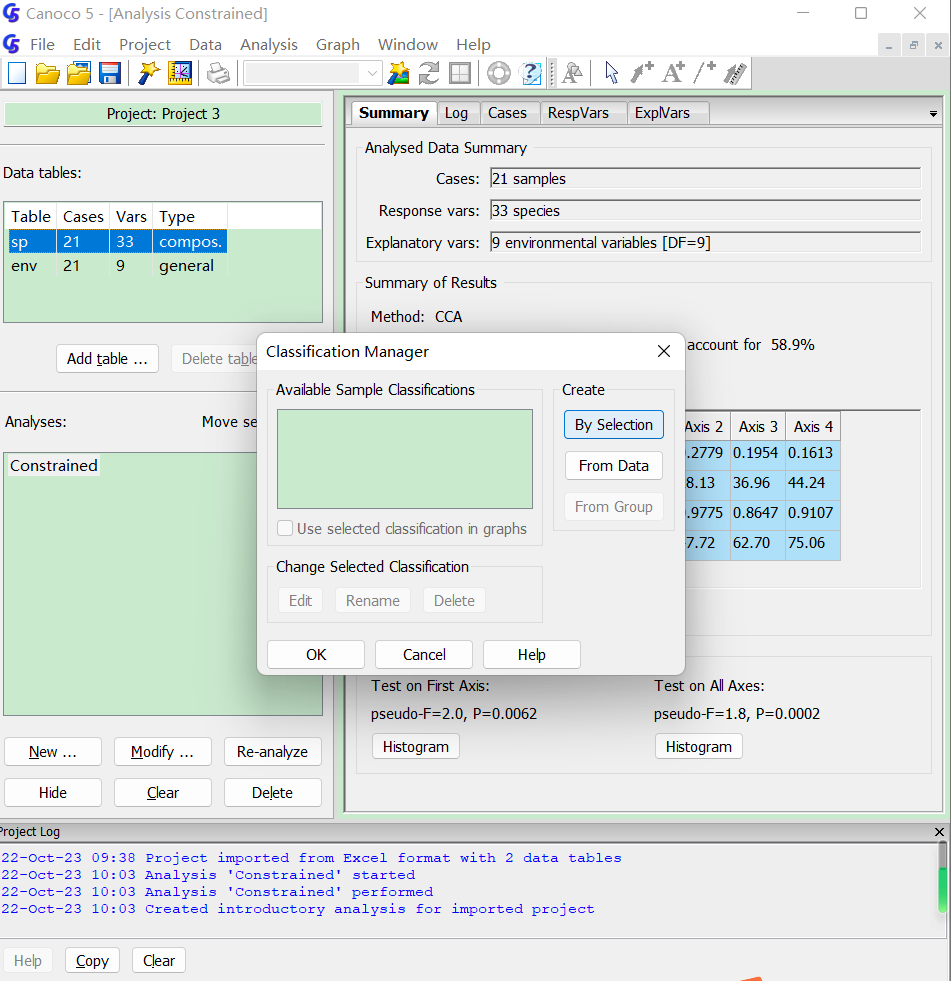


1. According to the left figure of TWINSPAN results, add seven associations (assoc. 1 to 7) and select its members in each association. Press the *OK* button.
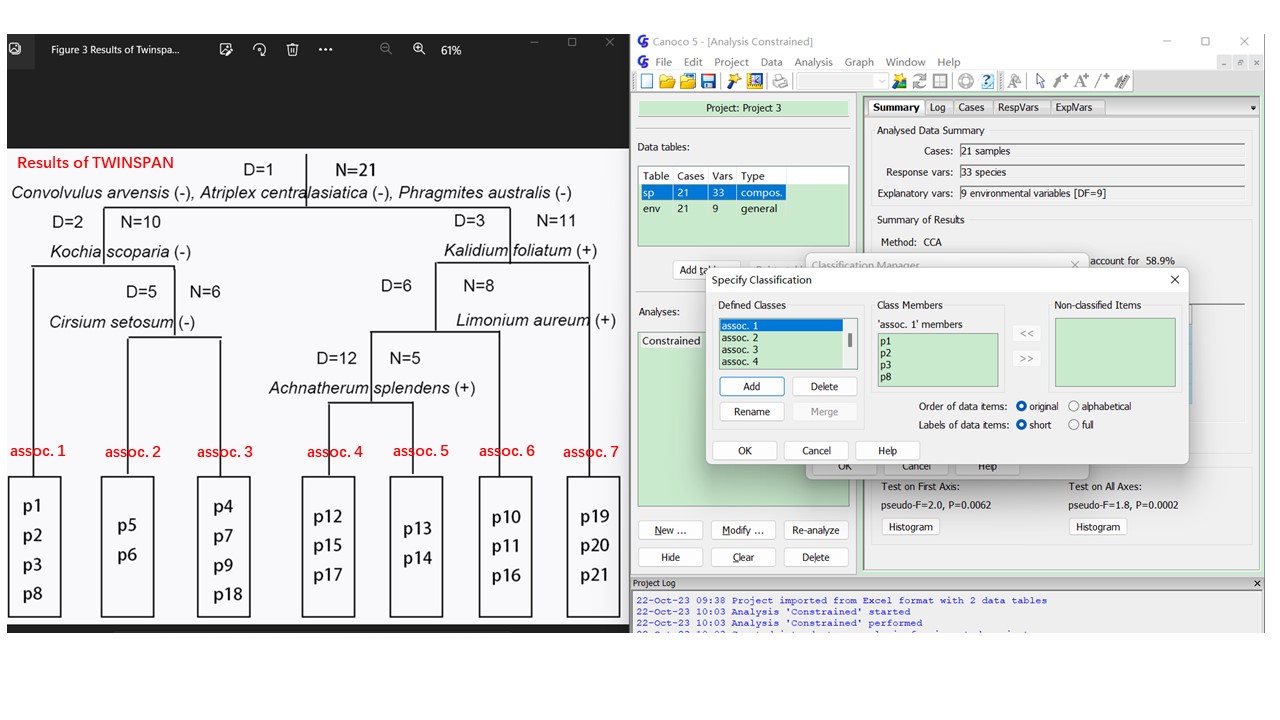

2. In the Classification Manager dialog box, check the option (*Use selected classification in graphs*) and press OK button again.


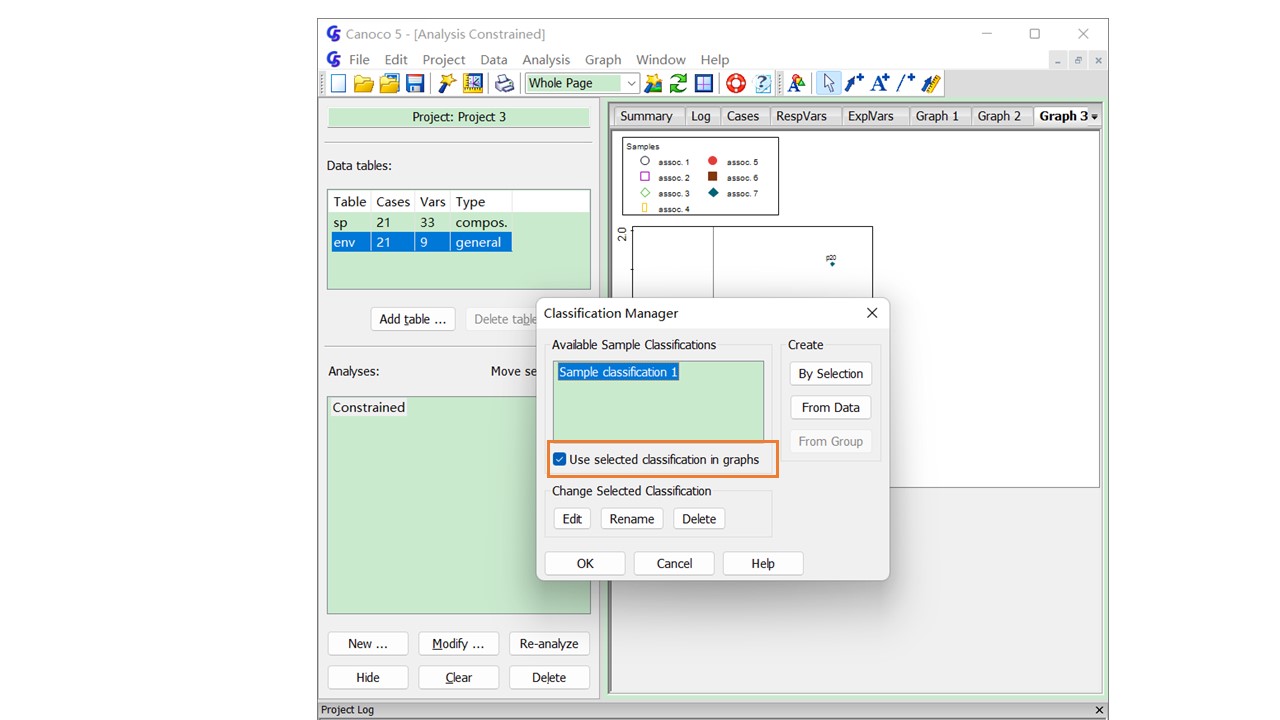


1. Plot ordination diagram of plots by selecting Graph | Scatterplots | Samples.


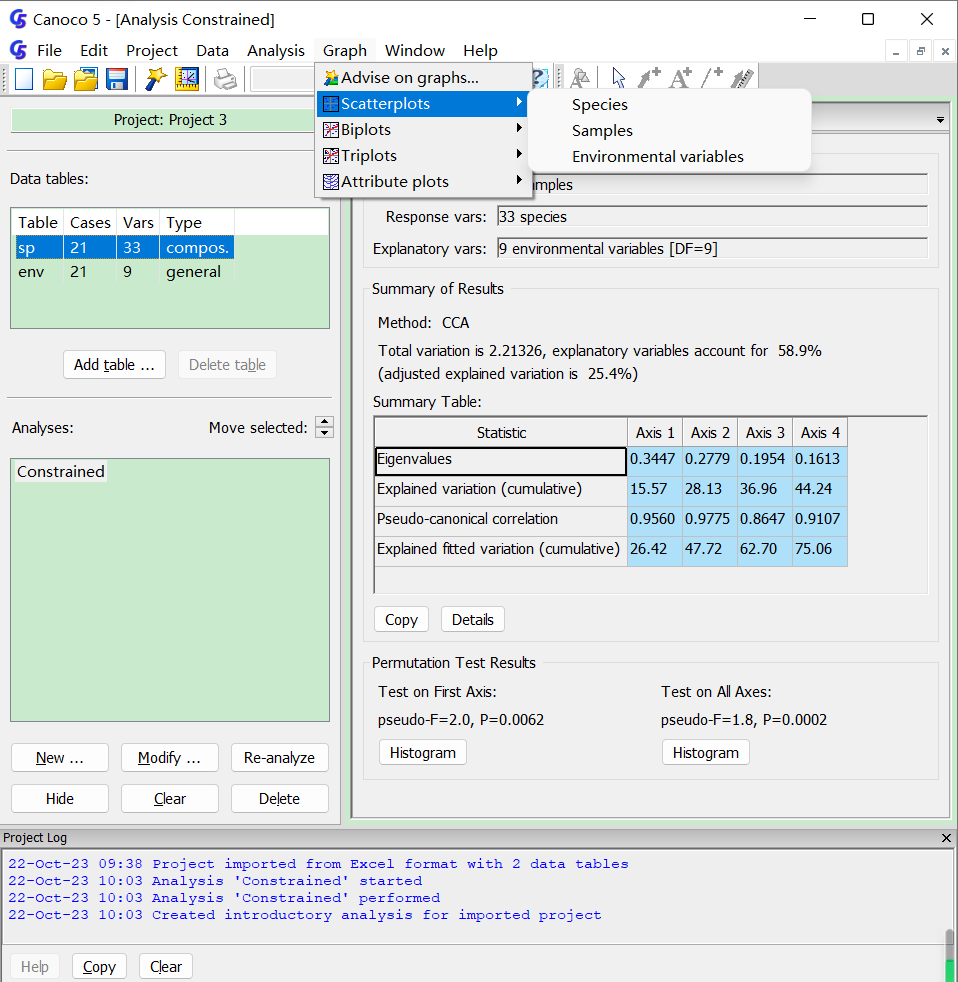


1.
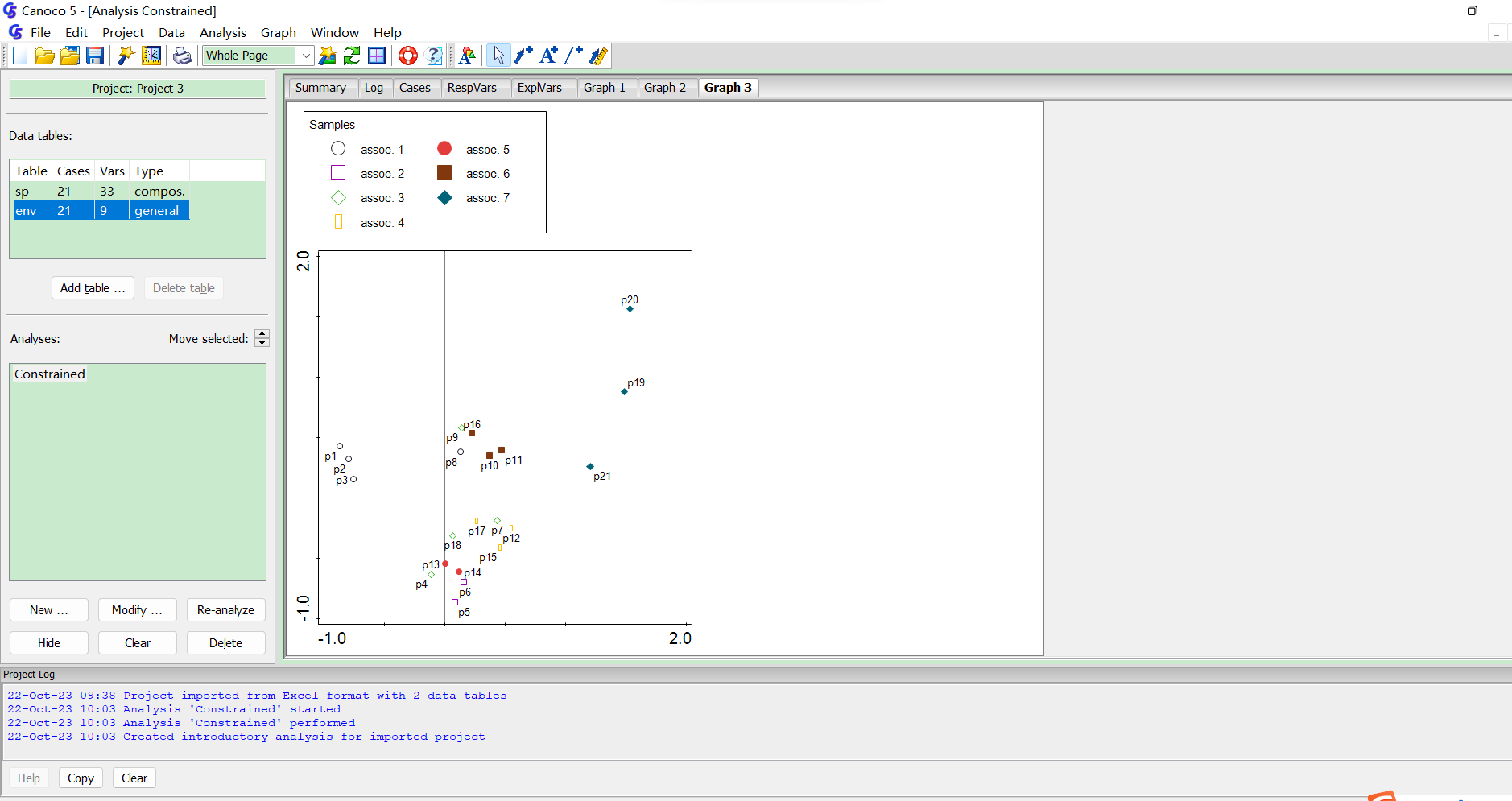
The resulting ordination diagram of plots is shown below.
2. Plot ordination diagram of soil variables by selecting Graph | Scatterplots | Environmental Variables.


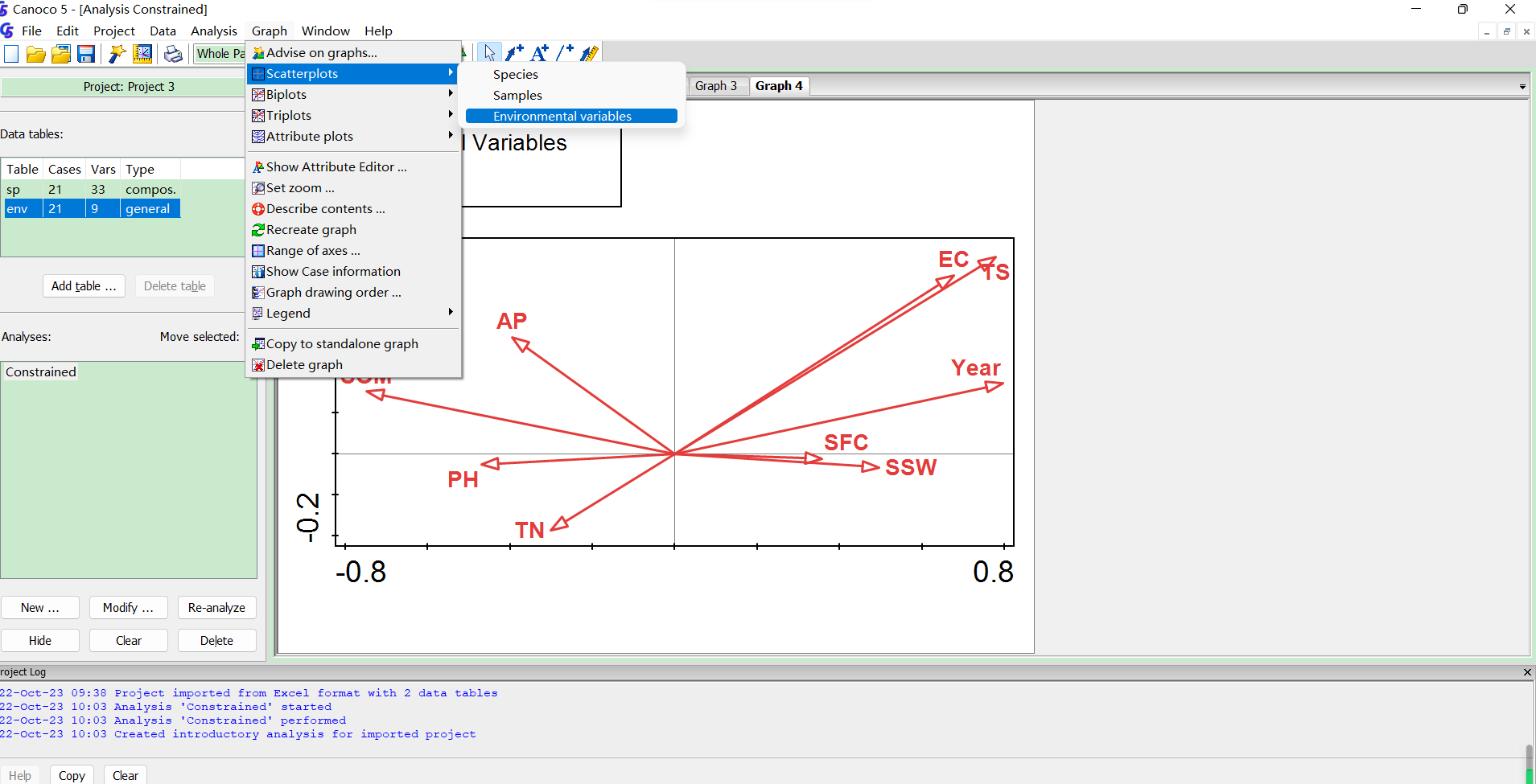


1. The resulting ordination plot of soil variables is shown below.


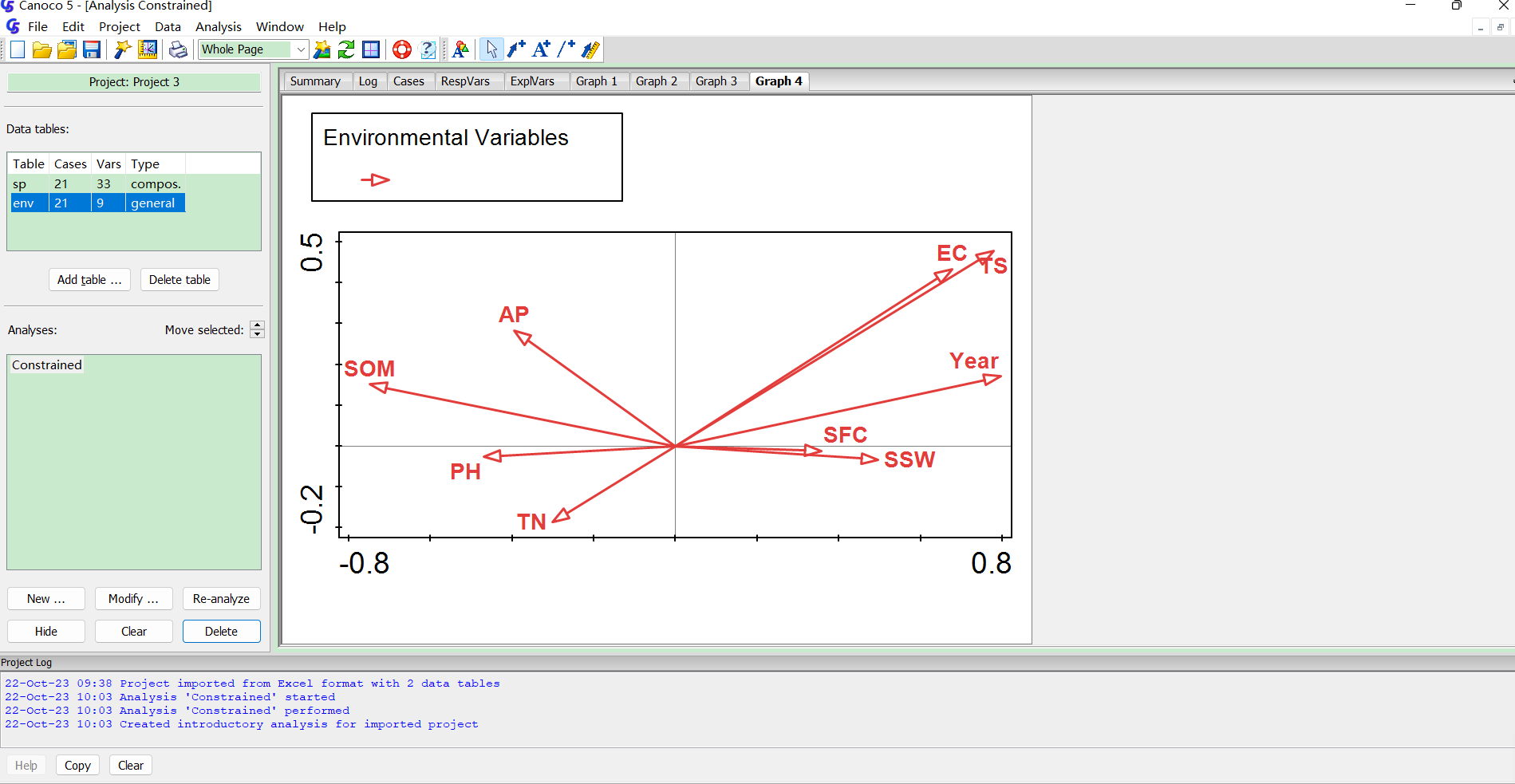

Supplement: Supplemental Information 2 — The metadata, raw data, analysis workflow, and result of: 1TWINSPAN-workflow, 2CCA-workflow, 3GAM-species response curves-workflow, and 4K-W test of plant diversity-workflow. [file peerj-12-17627-s002.zip › workflow/2CCA-workflow/2-4CCA-workflow.docx]
