# Supplementary material for: Exploring main soil drivers of vegetation succession in abandoned croplands of Minqin Oasis, China
Source: PeerJ. 2024 Jul 5;12:e17627. doi: 10.7717/peerj.17627 (PMC11229685; doi:10.7717/peerj.17627)
Supplement: Supplemental Information 2 — The metadata, raw data, analysis workflow, and result of: 1TWINSPAN-workflow, 2CCA-workflow, 3GAM-species response curves-workflow, and 4K-W test of plant diversity-workflow. [file peerj-12-17627-s002.zip › workflow/2CCA-workflow/2-5Simple and Conditional term effects workflow.docx]

Forward selection of the soil variables

1、Open canoco 5 software, and according to the CCA workflow, input the two matrix files “soil.xlsx” and “species1.xlsx” as source data. A dialog box of Introductory Analysis is shown below. Press the *Yes* button.


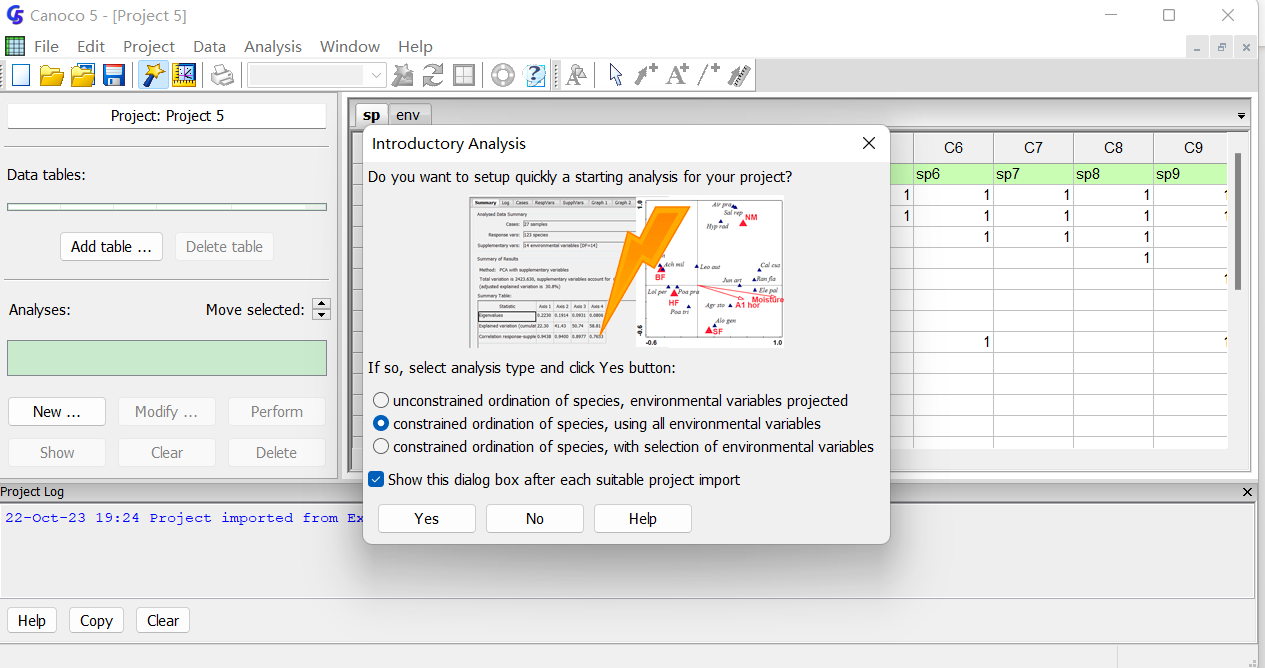


2、In this dialog box, check the option of Downweight rare species. then press the Next button.


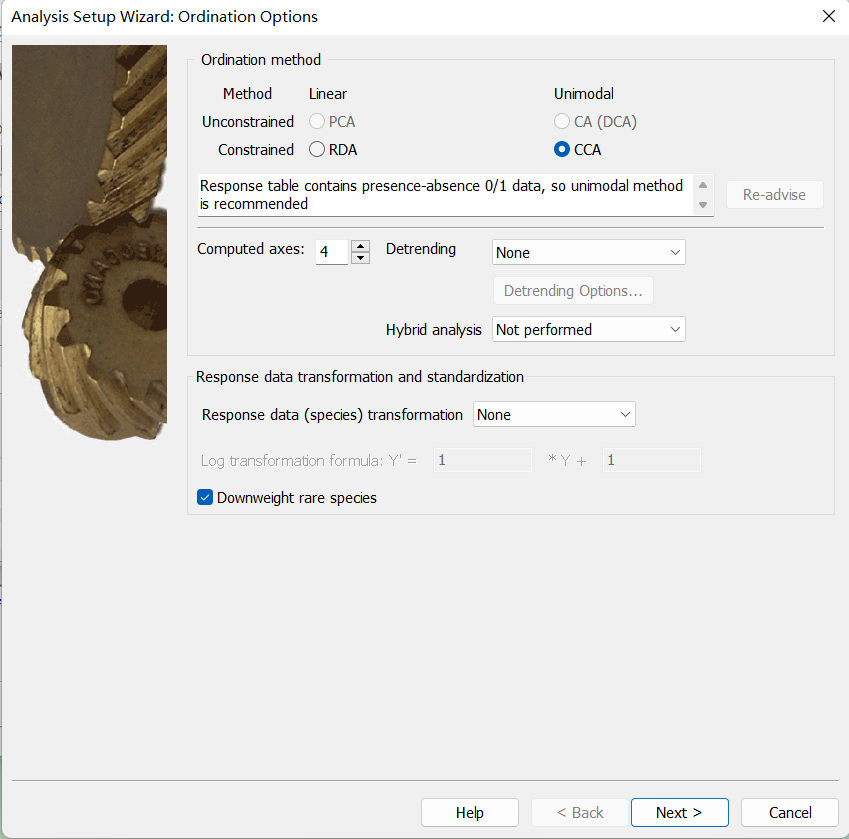


3、In this dialog box, select the option of *Summarize effects of expl. variables* and type “9999” as Number of permutations, then press the *Next* button and close the setup wizard on the following page with the *Finish* button.


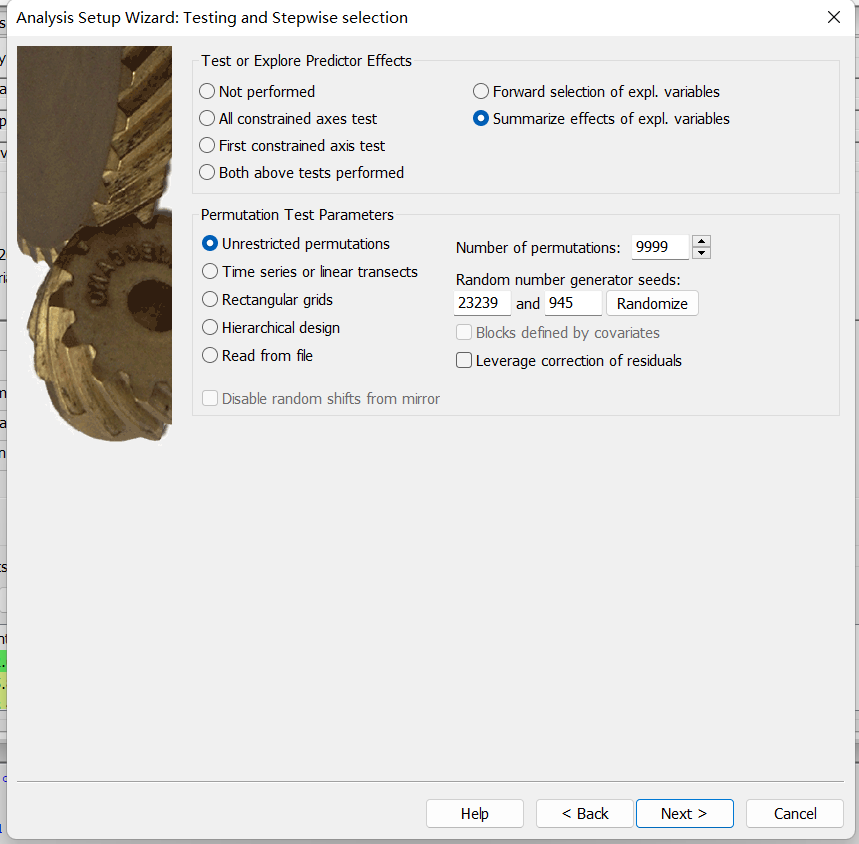


4、This page shows the result of Simple and Conditional term effects in the *Summary* tab.


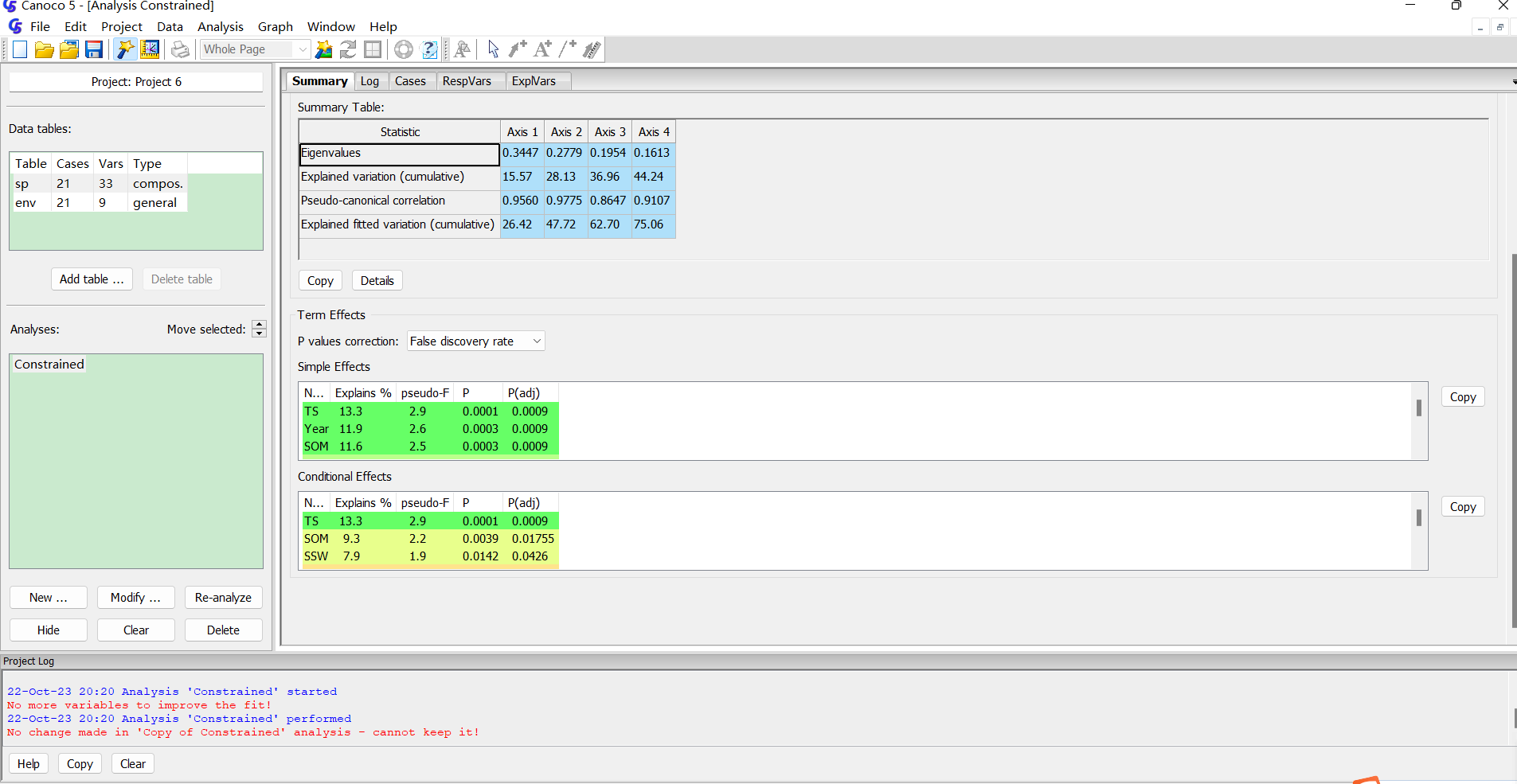


5、The data of the following table was calculated.

**Table 3:**

**Simple and conditional effects of explanatory variables**

| Name | Explains % | | pseudo-F | | *P* | | *P*(adj) |
| --- | --- | --- | --- | --- | --- | --- | --- |
| Simple term effects: | |  | |  | |  | |
| TS | 13.3 | | 2.9 | | 0.0001 | | 0.0009 |
| Year | 11.9 | | 2.6 | | 0.0003 | | 0.0009 |
| SOM | 11.6 | | 2.5 | | 0.0003 | | 0.0009 |
| EC | 11 | | 2.4 | | 0.0009 | | 0.00202 |
| SSW | 9 | | 1.9 | | 0.0109 | | 0.01962 |
| AP | 7.6 | | 1.6 | | 0.0553 | | 0.08229 |
| SFC | 7.4 | | 1.5 | | 0.064 | | 0.08229 |
| pH | 5.5 | | 1.1 | | 0.3143 | | 0.35359 |
| TN | 5.1 | | 1 | | 0.4172 | | 0.4172 |
| Conditional term effects: | |  | |  | |  | |
| TS | 13.3 | | 2.9 | | 0.0001 | | 0.0009 |
| SOM | 9.3 | | 2.2 | | 0.0039 | | 0.01755 |
| SSW | 7.9 | | 1.9 | | 0.0142 | | 0.0426 |
| Year | 6.7 | | 1.7 | | 0.0389 | | 0.0765 |
| SFC | 5.8 | | 1.5 | | 0.083 | | 0.1245 |
| TN | 6.1 | | 1.7 | | 0.0425 | | 0.0765 |
| EC | 3.3 | | 0.9 | | 0.5589 | | 0.5946 |
| AP | 3.4 | | 0.9 | | 0.5301 | | 0.5946 |
| pH | 3.1 | | 0.8 | | 0.5946 | | 0.5946 |

Note: *P*, level of significance of explanatory variables. *P*(adj), adjusted level of significance of explanatory variables by using the false discovery rate (FDR) approach. SOM, soil organic matter. AP, available phosphorus. EC, electrical conductivity. TN, total nitrogen. SSW, saturated soil water content. SFC, soil field capacity. TS, total salinity. Year, years of land abandonment.
