# Supplementary material for: Exploring main soil drivers of vegetation succession in abandoned croplands of Minqin Oasis, China
Source: PeerJ. 2024 Jul 5;12:e17627. doi: 10.7717/peerj.17627 (PMC11229685; doi:10.7717/peerj.17627)
Supplement: Supplemental Information 2 — The metadata, raw data, analysis workflow, and result of: 1TWINSPAN-workflow, 2CCA-workflow, 3GAM-species response curves-workflow, and 4K-W test of plant diversity-workflow. [file peerj-12-17627-s002.zip › workflow/2CCA-workflow/2-7Simple and Conditional term effects-result.docx]

======================

05-Nov-23 14:54 Performing analysis 'Constrained-3' with 1 step

------------------------------------------------

CANOCO Engine Version 5.1 April 2013, written by Cajo J.F. Ter Braak

(C) 1988 - 2013 Biometris - quantitative methods in the life and earth sciences

Plant Research International, Wageningen University and Research Centre

Box 100, 6700 AC Wageningen, The Netherlands

Downweighting rare response variables

Number of active cases: 21

Number of supplementary cases: 0

Total inertia in response data = 2.21326

N name (weighted) mean stand. dev. inflation factor

1 SOM 1.1381 0.2210 8.3658

2 AP 2.2258 0.3873 4.4436

3 pH 7.8702 0.1754 3.6136

4 EC 1552.0575 1721.4377 12.8813

5 TN 0.0619 0.0118 8.9984

6 SSW 35.6076 3.4305 10.7675

7 SFC 30.9210 2.9571 9.3621

8 TS 0.6791 0.6080 22.9807

9 Year 8.5587 7.7117 9.0354

* Check on influence in explanatory / covariate data *

The following case(s) have extreme values

Case Explanatory Covariate + Explanatory space

variable influence influence influence

p19 EC 5.0x

p20 EC 5.2x

* End of check *

* Start of forward selection of explanatory variables *

Variable 'TS' selected (adds 23.6 % to explanatory power)

Variable 'SFC' selected (adds 13.2 % to explanatory power)

Variable 'SSW' selected (adds 15.9 % to explanatory power)

Variable 'AP' selected (adds 13.7 % to explanatory power)

Variable 'EC' selected (adds 8.91 % to explanatory power)

Variable 'SOM' selected (adds 8.57 % to explanatory power)

Variable 'Year' selected (adds 8.03 % to explanatory power)

Variable 'pH' selected (adds 4.52 % to explanatory power)

Variable 'TN' selected (adds 3.59 % to explanatory power)

No more variables to improve the fit!

N name (weighted) mean stand. dev. inflation factor

1 SOM 1.1381 0.2210 8.3658

2 AP 2.2258 0.3873 4.4436

3 pH 7.8702 0.1754 3.6136

4 EC 1552.0575 1721.4377 12.8813

5 TN 0.0619 0.0118 8.9984

6 SSW 35.6076 3.4305 10.7675

7 SFC 30.9210 2.9571 9.3621

8 TS 0.6791 0.6080 22.9807

9 Year 8.5587 7.7117 9.0354

Iteration report for axis 1

Residual 0.046336 at iteration 0

Residual 0.002635 at iteration 1

Residual 0.000143 at iteration 2

Residual 0.000009 at iteration 3

Residual 0.000001 at iteration 4

Eigenvalue 0.33529

Iteration report for axis 2

Residual 0.035553 at iteration 0

Residual 0.003179 at iteration 1

Residual 0.000043 at iteration 2

Residual 0.000001 at iteration 3

Residual 0.000000 at iteration 4

Eigenvalue 0.27122

Iteration report for axis 3

Residual 0.018541 at iteration 0

Residual 0.001742 at iteration 1

Residual 0.000516 at iteration 2

Residual 0.000046 at iteration 3

Residual 0.000014 at iteration 4

Residual 0.000001 at iteration 5

Residual 0.000000 at iteration 6

Eigenvalue 0.17989

Iteration report for axis 4

Residual 0.042109 at iteration 0

Residual 0.000940 at iteration 1

Residual 0.000008 at iteration 2

Residual 0.000000 at iteration 3

Eigenvalue 0.16422

* Weighted correlation matrix (weight = case total) *

Resp Ax1 1.0000

Resp Ax2 -0.0437 1.0000

Resp Ax3 0.0735 -0.0446 1.0000

Resp Ax4 0.0503 0.0123 0.1418 1.0000

Expl Ax1 0.9519 0.0000 -0.0000 0.0000 1.0000

Expl Ax2 0.0000 0.9637 0.0000 0.0000 0.0000 1.0000

Expl Ax3 0.0000 -0.0000 0.8465 0.0000 -0.0000 0.0000 1.0000

Expl Ax4 -0.0000 -0.0000 -0.0000 0.8829 -0.0000 0.0000 -0.0000

SOM -0.4748 0.0247 0.0329 -0.4651 -0.4988 0.0256 0.0389

AP -0.3159 0.3502 -0.3862 -0.4021 -0.3319 0.3634 -0.4562

pH -0.4529 0.0354 -0.2209 0.1170 -0.4757 0.0367 -0.2609

EC 0.7016 0.3246 0.1319 -0.1510 0.7370 0.3368 0.1558

TN -0.2818 -0.1300 -0.2231 -0.3529 -0.2961 -0.1349 -0.2635

SSW 0.5363 -0.0413 0.1956 -0.4940 0.5634 -0.0429 0.2310

SFC 0.3157 0.1624 0.4162 -0.3882 0.3316 0.1685 0.4917

TS 0.8075 0.3489 0.1475 -0.1272 0.8483 0.3620 0.1743

Year 0.7860 0.0413 0.2602 -0.0589 0.8257 0.0429 0.3074

Resp Ax1 Resp Ax2 Resp Ax3 Resp Ax4 Expl Ax1 Expl Ax2 Expl Ax3

Expl Ax4 1.0000

SOM -0.5268 1.0000

AP -0.4554 0.4947 1.0000

pH 0.1326 0.2711 0.4700 1.0000

EC -0.1710 -0.4931 -0.3512 -0.6641 1.0000

TN -0.3997 0.8792 0.4689 0.2330 -0.4961 1.0000

SSW -0.5595 -0.1522 -0.0581 -0.7116 0.7104 -0.1491 1.0000

SFC -0.4397 -0.1885 -0.0943 -0.6387 0.6765 -0.3188 0.8899

TS -0.1440 -0.5113 -0.2195 -0.5799 0.9339 -0.4678 0.7247

Year -0.0667 -0.5781 -0.5558 -0.5694 0.8156 -0.5299 0.5585

Expl Ax4 SOM AP pH EC TN SSW

SFC 1.0000

TS 0.6798 1.0000

Year 0.5077 0.8491 1.0000

SFC TS Year

**** Summary ****

Axes 1 2 3 4 Total inertia

Eigenvalues : 0.335 0.271 0.180 0.164 2.213

Pseudo-canonical correlations : 0.952 0.964 0.847 0.883

Cumulative percentage variance

of response data : 15.1 27.4 35.5 43.0

of fitted response data : 26.9 48.6 63.0 76.1

Sum of all eigenvalues: 2.2133

Sum of all canonical eigenvalues: 1.2485

All four eigenvalues reported above are canonical and correspond to axes that

are constrained by the explanatory variables.

Cannot offer creation of 'classified sample diagram' suggested by Canoco Adviser, because the predictors are not of right type or count

Cannot offer creation of 'species pie symbol diagram' suggested by Canoco Adviser, because the predictors are not of right type or count

Cannot offer creation of 'star plot of sample classes' suggested by Canoco Adviser, because the predictors are not of right type or count

05-Nov-23 16:34 Created ordination diagram 'Samples scatter plot'

and stored it as Graph 1 in analysis notebook

Graph exported into file D:\׷\6-΄\Peer J\figures\Figure 4 CCA ordination diagram of 21 plots1.png in PNG Format

Graph exported into file D:\׷\6-΄\Peer J\figures\Figure 4 CCA ordination diagram of 21 plots1.png in PNG Format

Graph exported into file D:\׷\6-΄\Peer J\figures\Figure 4 CCA ordination diagram of 21 plots1.png in PNG Format

Analysis 'Constrained-3'

Simple Term Effects:

Name Explains % pseudo-F P P(adj)

TS 13.3 2.9 0.0001 0.0009

Year 11.9 2.6 0.0003 0.00135

EC 11.0 2.4 0.0018 0.0054

SSW 9.0 1.9 0.0085 0.01913

AP 7.6 1.6 0.0574 0.08859

SFC 7.4 1.5 0.0689 0.08859

SOM 7.3 1.5 0.0652 0.08859

pH 5.5 1.1 0.3143 0.35359

TN 5.1 1.0 0.4172 0.4172

Analysis 'Constrained-3'

Conditional Term Effects:

Name Explains % pseudo-F P P(adj)

TS 13.3 2.9 0.0001 0.0009

SFC 7.4 1.7 0.0372 0.0837

SSW 9.0 2.2 0.0078 0.0351

AP 7.7 2.0 0.0188 0.0564

EC 5.0 1.3 0.1814 0.31243

SOM 4.8 1.3 0.2095 0.31243

Year 4.5 1.2 0.243 0.31243

pH 2.5 0.7 0.7781 0.87536

TN 2.0 0.5 0.9053 0.9053
