# Supplementary material for: Exploring main soil drivers of vegetation succession in abandoned croplands of Minqin Oasis, China
Source: PeerJ. 2024 Jul 5;12:e17627. doi: 10.7717/peerj.17627 (PMC11229685; doi:10.7717/peerj.17627)

**File specification**: There are five files in the folder.

1. “3-1read me-metadata.docx” is an instruction file.

2. Both “3-2species data.xlsx” and “3-3soil data.xlsx” are data files. The data format and the meaning are the same as CCA analysis, please refer to the file of “2-1read me-metadata.docx” in the folder of CCA-workflow if you need.

3. “3-4GAM-workflow.doc” is a workflow file, and it shows us how to perform the GAM analysis and plot species response curves in the software of Canoco 5.0.

4. “3-5result-GAM.xlsx” is a result file.


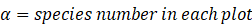

Supplement: Supplemental Information 2 — The metadata, raw data, analysis workflow, and result of: 1TWINSPAN-workflow, 2CCA-workflow, 3GAM-species response curves-workflow, and 4K-W test of plant diversity-workflow. [file peerj-12-17627-s002.zip › workflow/3GAM-species response curves-workflow/3-1read me-metadata.docx]
