# Supplementary material for: Exploring main soil drivers of vegetation succession in abandoned croplands of Minqin Oasis, China
Source: PeerJ. 2024 Jul 5;12:e17627. doi: 10.7717/peerj.17627 (PMC11229685; doi:10.7717/peerj.17627)

1. Run R software, and open a program package of “Kruskal-Wallis test.R” select File | Open File.


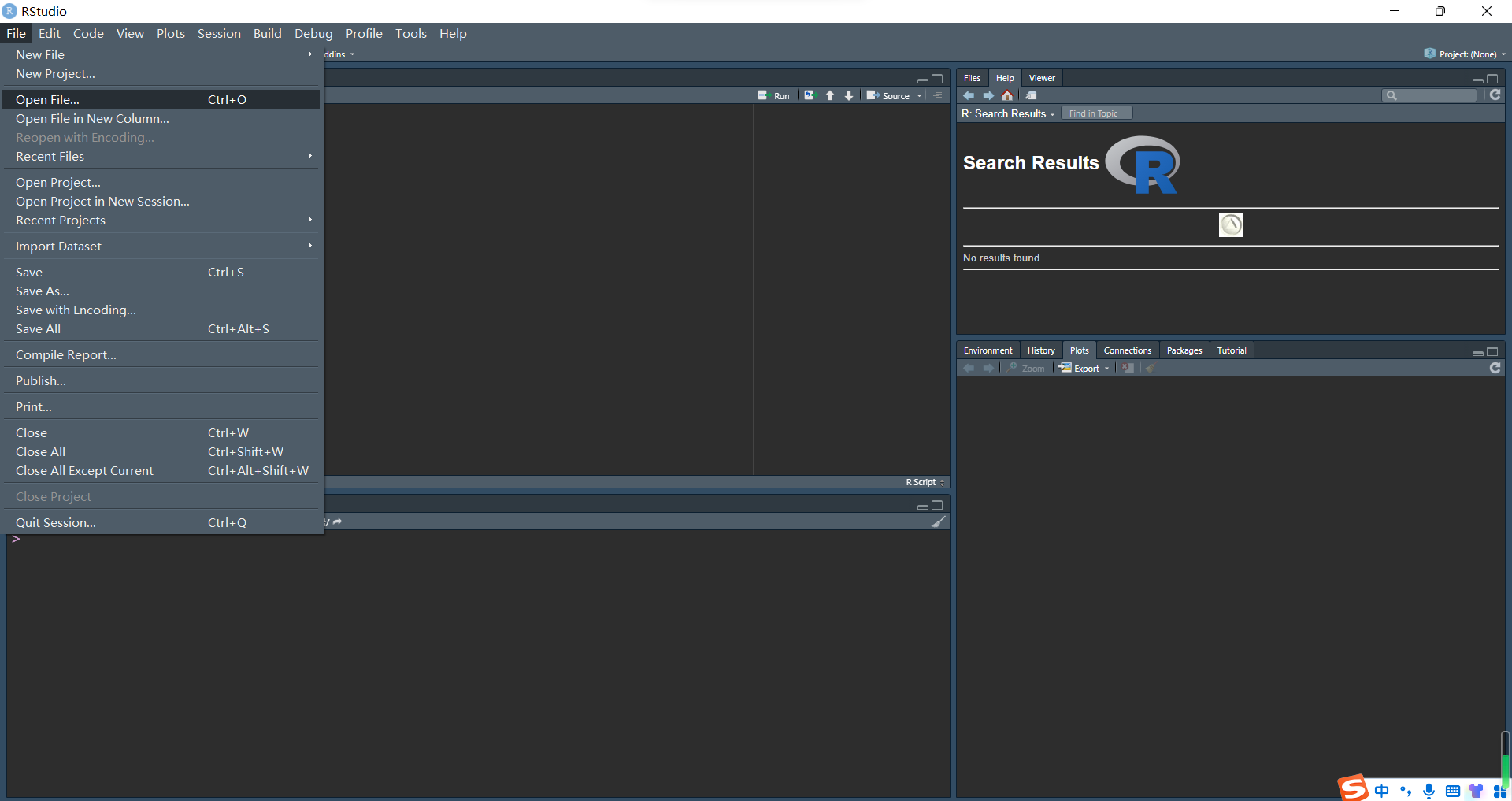


2. On the top left of the screen, select all the code and press the *Run* button.


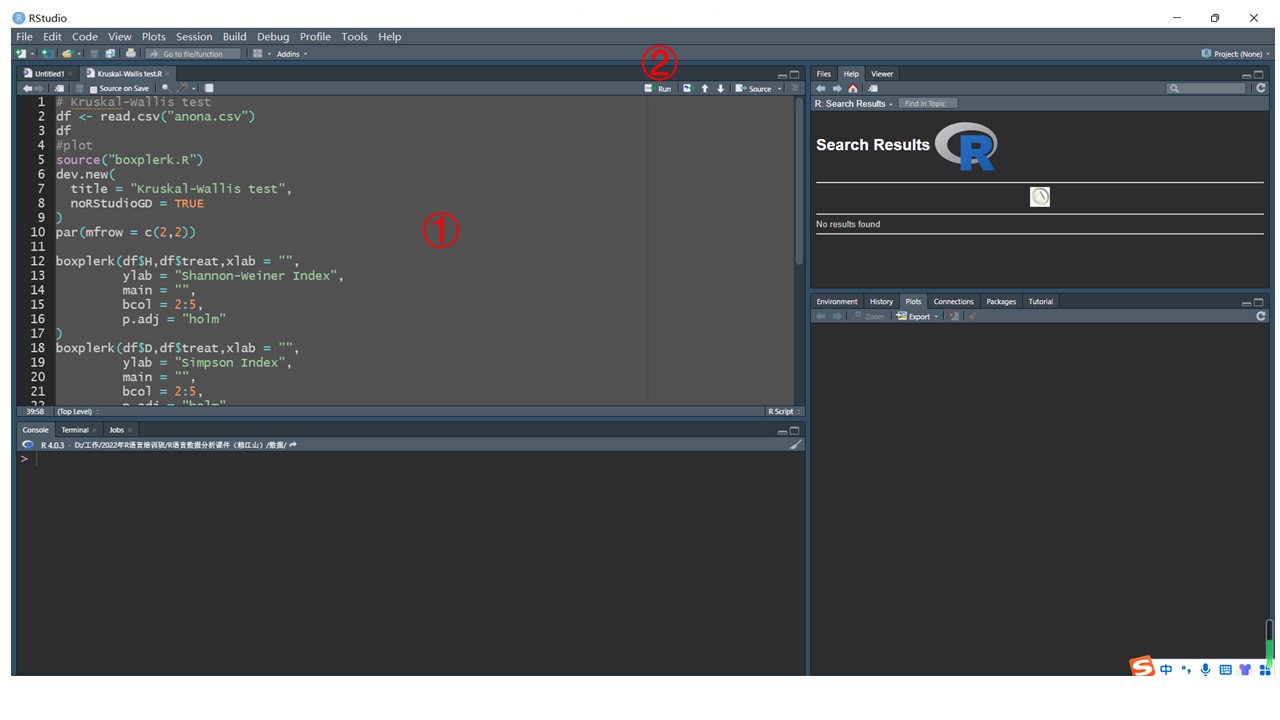


3. The result is on the bottom left of the screen, and the figure is on the right.


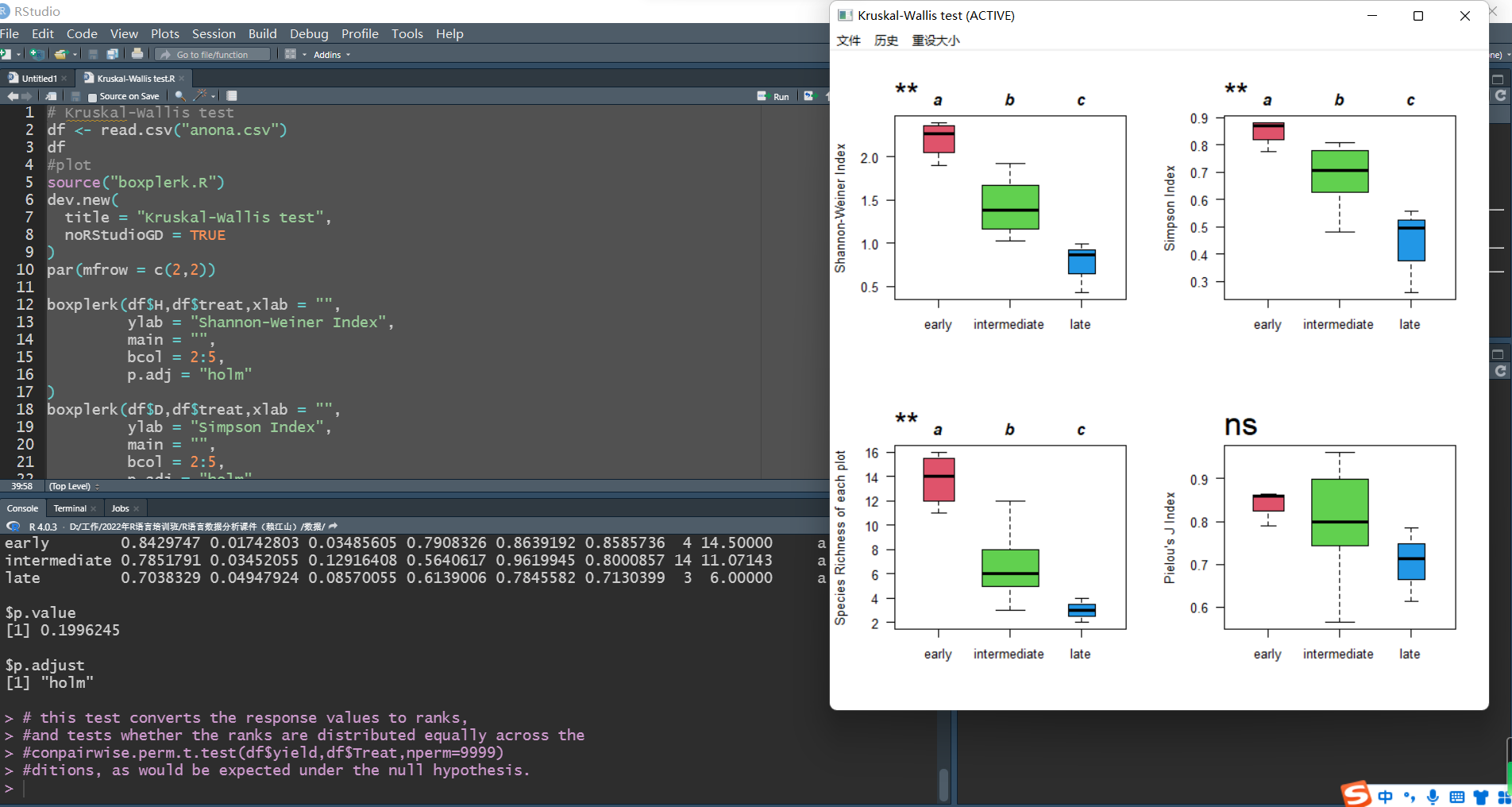

Supplement: Supplemental Information 2 — The metadata, raw data, analysis workflow, and result of: 1TWINSPAN-workflow, 2CCA-workflow, 3GAM-species response curves-workflow, and 4K-W test of plant diversity-workflow. [file peerj-12-17627-s002.zip › workflow/4K-W test of plant diversity-workflow/4-2K-W test of plant diversity-workflow.docx]
