# Supplementary material for: Exploring main soil drivers of vegetation succession in abandoned croplands of Minqin Oasis, China
Source: PeerJ. 2024 Jul 5;12:e17627. doi: 10.7717/peerj.17627 (PMC11229685; doi:10.7717/peerj.17627)

**Tips:** At the beginning, please copy this folder in your computer, and perform Kruskal-Wallis test of plant diversity according to the instruction of the file of “4-2K-W test of plant diversity-workflow.docx”, then the Figure 5 in our article will be drawn soon.


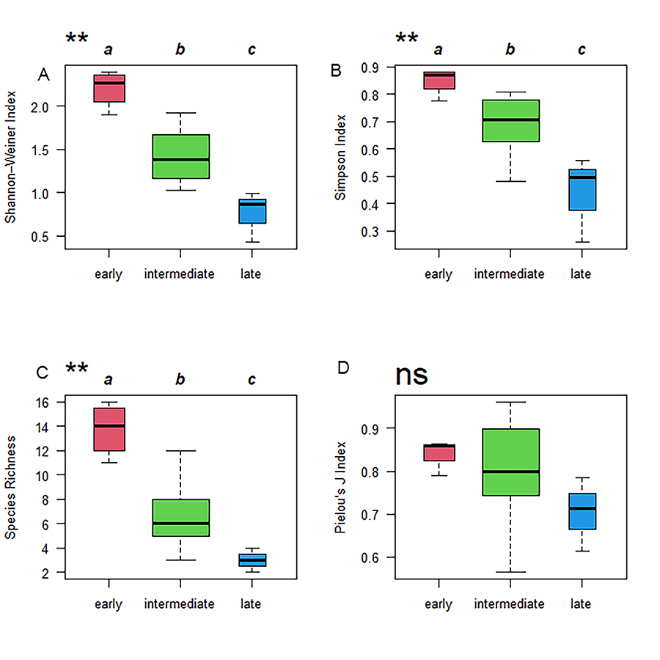


**File specification**: There are five files in the folder.

1. “4-1Kruskal-Wallis test.R” is a program package. After you run this program package in R software, you can perform Kruskal-Wallis test of plant diversity and draw the box plot of the result at the same time.

2. “4-2K-W test of plant diversity-workflow.docx” is a workflow file, and it shows us how to perform the Kruskar-Wallis test of plant diversity, and draw the box plots.

3. “4-3plant diversity data.csv” is a data file. It is based on a 4 column × 21 row (4 plant diversities × 21 plots) matrix data. These plots are divided into three successional stages by using CCA and TWINSPAN methods (labelled by early, intermediate, and late). The early successional stage contains four plots (labelled by p1, p2, p3, p8), the intermediate successional stage contains fourteen plots (labelled by p4-7, p9, p10-18), and the late successional stage contains three plots (labelled by p19-21). The meaning of the four plant diversity indexes is in the following. “H” represents Shannon-Weiner Index, “D” represents Simpson Index, “N” represents species richness, and “E” represents Pielou’s index.

4. “4-4read me-metadata.docx” is an instruction file.

5. “4-5results-Kruskar-Wallis test.txt” is a result file.


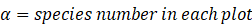

Supplement: Supplemental Information 2 — The metadata, raw data, analysis workflow, and result of: 1TWINSPAN-workflow, 2CCA-workflow, 3GAM-species response curves-workflow, and 4K-W test of plant diversity-workflow. [file peerj-12-17627-s002.zip › workflow/4K-W test of plant diversity-workflow/4-4read me-metadata.docx]
