# Supplementary material for: Exploring main soil drivers of vegetation succession in abandoned croplands of Minqin Oasis, China
Source: PeerJ. 2024 Jul 5;12:e17627. doi: 10.7717/peerj.17627 (PMC11229685; doi:10.7717/peerj.17627)
Supplement: Supplemental Information 2 — The metadata, raw data, analysis workflow, and result of: 1TWINSPAN-workflow, 2CCA-workflow, 3GAM-species response curves-workflow, and 4K-W test of plant diversity-workflow. [file peerj-12-17627-s002.zip › workflow/1TWINSPAN-workflow/1-4TWINSPAN-workflow.doc]

1. Open the software of PC-ORD 5.0, and the window is open.

2. Input raw data.
(1) Input a matrix file (“species.xls”) by selecting File|Import Matrix|Main Matrix|Excel Spreadsheet.

(2) The raw data file has been inputted in the software of PC-ORD 5.0.

3. TWINSPAN analysis.
(1) Perform TWINSPAN analysis by selecting Groups | TWINSPAN, and press “OK” twice.

(2) The result file is appearing in the window after calculation.
