# Supplementary material for: Exploring main soil drivers of vegetation succession in abandoned croplands of Minqin Oasis, China
Source: PeerJ. 2024 Jul 5;12:e17627. doi: 10.7717/peerj.17627 (PMC11229685; doi:10.7717/peerj.17627)
Supplement: Supplemental Information 2 — The metadata, raw data, analysis workflow, and result of: 1TWINSPAN-workflow, 2CCA-workflow, 3GAM-species response curves-workflow, and 4K-W test of plant diversity-workflow. [file peerj-12-17627-s002.zip › workflow/3GAM-species response curves-workflow/3-4GAM-workflow.doc]

**GAM-species response curves**

1. Open the software of Canoco 5.0, and perform CCA analysis according to the CCA workflow. A page of CCA results is shown below.

2. **Set seven dominant species as a group.**
(1) Select Project | Groups | of species to set a group of dominant species.

(2) After press the *By Selection* button, a new dialog box is shown, where you can choose seven dominant species from the right to the left, and press the *OK* button twice in the two dialog boxex.

3. **Perform GAM-species response curves analysis**
(1) Select Graph | Attribute plots | Species response curves, and then a new dialog box will be shown.

(2) At the new dialog box of the *Response Cures Plot*, specify *GAM* as the Model type in the upper right corner of this dialog, select the name (*Dominant species*) of the group you just created as the Response group, and select Environmental variables in a dropdown list, then select one variable as the *Predictor variable* once. Before leaving this dialog, specify options for GAM models in the dialog shown by the *Model Options* button.

(3) After click the *Model Options* button, a new dialog of GAM Options is shown. Specify the value 4 in the *Term Smoothness* field, choose *Poisson* distribution as the *Response Distribution* in the middle of the dialog, check the option of the *Stepwise selection using AIC* (Akaike Imformation Criterion), and preceding dialog with the *OK* buttons, the regression model is fitted and the results are summarised in another dialog box. When you choose another environmental variable, you can repeat (2) and (3) to perform GAM model and draw species response curves.

(4) This page shows us the results and the species response curves being fitted by GAM model.
